# Logistic‐growth models measuring density feedback are sensitive to population declines, but not fluctuating carrying capacity

**DOI:** 10.1002/ece3.10010

**Published:** 2023-04-26

**Authors:** Corey J. A. Bradshaw, Salvador Herrando‐Pérez

**Affiliations:** ^1^ Global Ecology, College of Science and Engineering Flinders University Adelaide South Australia Australia; ^2^ Australian Research Council Centre of Excellence for Australian Biodiversity and Heritage Wollongong New South Wales Australia; ^3^ Department of Biogeography and Global Change Museo Nacional de Ciencias Naturales, Spanish National Research Council (CSIC) Madrid Spain

**Keywords:** Australia, compensation, demographic rate, density dependence, megafauna, population size, stationarity, time series

## Abstract

Analysis of long‐term trends in abundance of animal populations provides insights into population dynamics. Population growth rates are the emergent interplay of *inter alia* fertility, survival, and dispersal. However, the density feedbacks operating on some vital rates (“component feedback”) can be decoupled from density feedbacks on population growth rates estimated using abundance time series (“ensemble feedback”). Many of the mechanisms responsible for this decoupling are poorly understood, thereby questioning the validity of using logistic‐growth models versus vital rates to infer long‐term population trends. To examine which conditions lead to decoupling, we simulated age‐structured populations of long‐lived vertebrates experiencing component density feedbacks on survival. We then quantified how imposed stochasticity in survival rates, density‐independent mortality (catastrophes, harvest‐like removal of individuals) and variation in carrying capacity modified the ensemble feedback in abundance time series simulated from age‐structured populations. The statistical detection of ensemble density feedback from census data was largely unaffected by density‐independent processes. Long‐term population decline caused from density‐independent mortality was the main mechanism decoupling the strength of component versus ensemble density feedbacks. Our study supports the use of simple logistic‐growth models to capture long‐term population trends, mediated by changes in population abundance, when survival rates are stochastic, carrying capacity fluctuates, and populations experience moderate catastrophic mortality over time.

## Graphical abstract

We simulated component density feedbacks on survival in age‐structured populations of long‐lived vertebrates and quantified how imposed density‐independent mortality and variation in carryingcapacity modified the ensemble density feedback strength estimated from logistic‐growth models to the simulated abundance time series. Catastrophic and proportional mortality eroded the effect of density‐dependent survival on ensemble‐feedback strength more strongly than variation in carrying capacity. Thus, phenomenological models offer a robust approach to capture the strength of density feedbacks from nonstationary census data when density‐independent mortality is low.

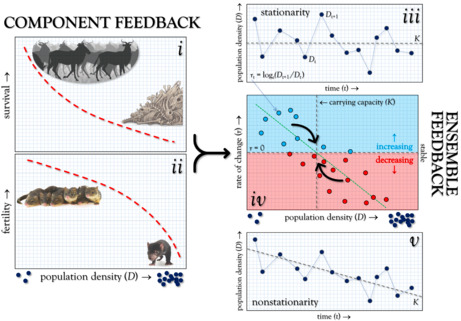

## 1 INTRODUCTION

Compensatory density feedback describes a population's ability to return to the environment's carrying capacity in response to an increase in population size (*sensu* Herrando‐Pérez et al., 2012b)—see the full glossary of terms in Table 1. Density feedback is a phenomenon driven by adjustments to individual fitness imposed by variation in per‐capita resource availability, dispersal, and associated trophic and social interactions, including competition, predation, and parasitism (Eberhardt et al., 2008; Herrando‐Pérez et al., 2012a; Matthysen, 2005). As survival and fertility rates ebb and flow over time, it is theoretically possible to detect the presence, and quantify the strength, of density feedbacks in population growth rates using abundance time series. Such “census data” results from populations monitored at semiregular intervals over a sufficient period relative to the generation length of the species under investigation (Brook & Bradshaw, 2006; Herrando‐Pérez et al., 2012a). There is now considerable evidence that survival and reproduction track population trends in many vertebrate (Eberhardt, 2002; Morrison et al., 2021; Owen‐Smith & Mason, 2005; Paradis et al., 2002; Pardo et al., 2017) and invertebrate (Bonsall & Benmayor, 2005; Ma, 2021; Marini et al., 2016; McGeoch & Price, 2005) species. Therefore, given the irreplaceable importance of long‐term monitoring of population size in applied ecology and conservation (Bonebrake et al., 2010; Di Fonzo et al., 2016; Herrando‐Pérez et al., 2012a; Micheli et al., 2020), assessing the strength of compensatory signals in censuses of population abundance remains an essential tool in the ecologist's toolbox (Hostetler & Chandler, 2015; Johnson et al., 2022; Ponciano et al., 2018; Rueda‐Cediel et al., 2015; Thibaut & Connolly, 2020).

**TABLE 1:** Glossary of terms used in the paper. Italicized, boldface terms in the definition column indicate terms defined elsewhere in the table.

Term	Definition	References
***Carrying capacity***	Maximum population density (commonly denoted *K*) a given environment can sustain indefinitely, so describing the equilibrium population density as determined by available resources. *K* can be exceeded, but only temporarily. Mathematically, *K* equates to the long‐term mean ***population density*** where the ***per capita* *rate of exponential population change*** (*r*) approaches zero	Berryman and Turchin (2001); Berryman (1999)
***Cohort***	Group of individuals that were born at the same time. Used to assign individuals to the same age class within a ***Leslie matrix*** including specific ***demographic rates***	Gotelli (2008)
***Compensation* (*compensatory*)**	A density feedback whereby population density is negatively correlated with population growth, fertility, survival or dispersal. Population density declines when the ***carrying capacity*** is exceeded and vice versa	Neave (1953)
***Component density feedback***	Density feedback compensating or depensating single ***demographic rates***	Herrando‐Pérez et al. (2012b)
***Demographic rate***	A measurable aspect of individual fitness expressed as a probability or a rate over a defined time period, including *survival* (probability of an individual surviving from time *t* to *t* + 1), *fertility* (number of offspring per female produced per unit time), and *dispersal* (number of individuals leaving a defined population per unit time)	Levin et al. (2008)
***Density feedback* (*~density dependence*** ^a^ **)**	When social and trophic interactions modify ***demographic rates*** and the resulting change in ***demographic rates*** alters ***population density***, “feeding” back to modify the intensity of those interactions	Berryman (1989); Berryman et al. (2002)
***Density‐feedback strength***	The degree to which a demographic rate or population rate of change varies with increasing or decreasing ***population density***. In the ***Ricker logistic*** model, this is measured as the slope of the ***compensatory*** (negative) relationship between the ***per capita* *rate of exponential population change*** and ***population density***	Brook and Bradshaw (2006); Doncaster (2008)
***Density independence***	Present or past population density not affecting per‐capita population growth rate and/or ***demographic rates***	Herrando‐Pérez et al. (2012b); Smith (1935)
***Depensation* (*depensatory*)**	A density feedback whereby population density is positively correlated with population growth, fertility, survival, or dispersal. It typically occurs at low population density far from carrying capacity, often referred to as an “Allee effect”	Courchamp et al. (1999); Neave (1953)
***Ensemble density feedback***	***Compensation*** or ***depensation*** acting on a population's overall growth rate, representing the sum of all ***component density feedbacks***	Herrando‐Pérez et al. (2012a); Münster‐Swendsen and Berryman (2005)
***Gompertz logistic***	Linear (***compensatory***), discrete‐time relationship between the ***per capita* *rate of exponential population change*** (*r*) and the natural logarithm of ***population density***	Doncaster (2008); Medawar (1940); Nelder (1961)
***Leslie matrix***	Also known as a population demographic matrix, it represents the probability of transitioning from one age class to the next (survival), and producing new individuals in the first age class (fertility)	Caswell (2001)
***Nonstationarity* (*nonstationary*)**	Occurs when ***density‐dependent*** and **‐*independent*** mechanisms generating fluctuations in ***population density*** themselves change through time	Dennis and Taper (1994); Turchin (2003)
***Per capita* *rate of exponential population change***	Often denoted *r*, this is the rate of population change calculated as the natural logarithm of the ratio of ***population densities*** at time *t* + 1 to *t*, where *r* = log_*e*_(*N*_*t* + 1_/*N*_*t*_). When *r* = 0, the population is stable; when *r* < 0, it declines; when *r* > 1, the population is increasing	Turchin (2003)
***Phenomenological***	Model describing the long‐term dynamics of population density (cycles, stability, instability) resulting from demographic processes	Herrando‐Pérez et al. (2012b)
***Population density***	Often denoted *N*, this is the number of individuals in a population per unit area; when the area under consideration does not shift through time, population size can replace density per se in dynamical models	Berryman (1999)
***Return time***	Time required for a population to return to ***carrying capacity*** following a disturbance	Berryman (1999)
***Ricker logistic***	Linear (***compensatory***), discrete‐time relationship between the ***per capita* *rate of exponential population change*** (*r*) and ***population density***	Doncaster (2008); Ricker (1958)
***Stationarity* (*stationary*)**	Opposite of ***nonstationarity***; a dynamical system where the mechanisms generating fluctuations in population size do not change with time	Dennis and Taper (1994); Turchin (2003)
***Stochastic***	Property of models estimating the probability of various outcomes while allowing for uncertainty in one or more parameters. In stable (***stationary***) systems, stochasticity is due to environmental factors; in chaotic systems, variability is caused by both internal (components of population structure like density feedbacks) and environmental factors	Sinclair and Pech (1996)
***Time series***	Estimates of population abundance monitored at semi‐regular intervals (e.g., years), collectively known as “census”	Knape and de Valpine (2012)
***Vital rate***	See *demographic rate*	

^a^ ***Density feedback*** should replace ***density dependence*** because, while used synonymously, the former abates conceptual and terminological confusion (Herrando‐Pérez et al., 2012b).

The family of self‐limiting population‐growth models including logistic growth curves (“phenomenological models” hereafter) (Eberhardt et al., 2008) use census data to quantify the net effect of population size *N* on the per capita rate of exponential population change *r* (Berryman & Turchin, 2001). Expressed as a proportional change in *N* between two time (*t*) steps (e.g., years or generations), the assumption is that the discrete‐time metric *r*_*t*_ = log_*e*_(*N*_*t* + 1_/*N*_*t*_) summarizes the combination or “ensemble” (Herrando‐Pérez et al., 2012a) of all “component” density feedbacks operating on survival, fertility, and dispersal (Münster‐Swendsen & Berryman, 2005). The problem is that population growth rates can be insensitive to variation in particular demographic rates (Battaile & Trites, 2013; Bürgi et al., 2015; Kolb et al., 2010). Thus, across 109 observed censuses of bird and mammal populations, the strength of “component density feedback” (on demographic rates) explained only <10% of the strength of “ensemble density feedback” (on population growth rate) using logistic models after controlling for time‐series length and species' body size (Herrando‐Pérez et al., 2012a). The potential reasons for such decoupling include observation error (Abadi et al., 2012; Knape & de Valpine, 2012), fluctuating age structure (Hoy et al., 2020), unequal contribution to density feedbacks by age‐structured individuals (Gamelon et al., 2016), shifting nonstationarity among vital rates (Layton‐Matthews et al., 2019), immigration (Lieury et al., 2015), spatial heterogeneity (Thorson et al., 2015), and environmental state shifts (Turchin, 2003; Wu et al., 2007).

Determining the partial effects of different underlying mechanisms responsible for the decoupling of component and ensemble density feedbacks is virtually impossible for population censuses of real species. This limitation occurs for two main reasons: (1) the multiple, density‐dependent and ‐independent mechanisms generating population fluctuations change themselves through time—a condition known as “nonstationarity” (*sensu* Turchin, 2003), and (2) the full set of those mechanisms is often unknown and/or not measured in wild populations. To build a fully controlled simulation environment that incorporated most mechanisms a priori determined to affect component‐ensemble decoupling, we built stochastic, age‐structured population models with known, component density feedbacks on survival. We imposed nonstationarity to population size via multiple demographic scenarios emulating density‐independent mortality and temporal variation in carrying capacity. We then simulated multiannual time series of abundance from those populations and used them to estimate the strength of ensemble density feedbacks by means of discrete‐time logistic‐growth models. Based on previous findings from censuses of real populations (Herrando‐Pérez et al., 2012a), our hypothesis is that certain types and magnitudes of nonstationarity should erode the ability of logistic models to capture the strength of, and evidence for, ensemble density feedbacks when component density feedbacks are operating.

Specifically, we simulated the dynamics of 21 long‐lived vertebrates from a range of taxonomic/functional groups and body sizes, adjusting component feedbacks in each case to elicit initially stable dynamics. Theoretically, the strength of the ensemble signal (density feedback on population growth rate) must track the strength of the component signal (density feedback on survival), if survival has a demographic impact, mediated by population size, on the long‐term population trends. To test our hypothesis, we then imposed nonstationarity to each time series in the form of two density‐independent processes (catastrophic and harvest‐like mortality; fluctuating carrying capacity) to assess the extent by which those processes mask the effect of the component signal on the ensemble signal.

## 2 METHODS

**FIGURE 1:**
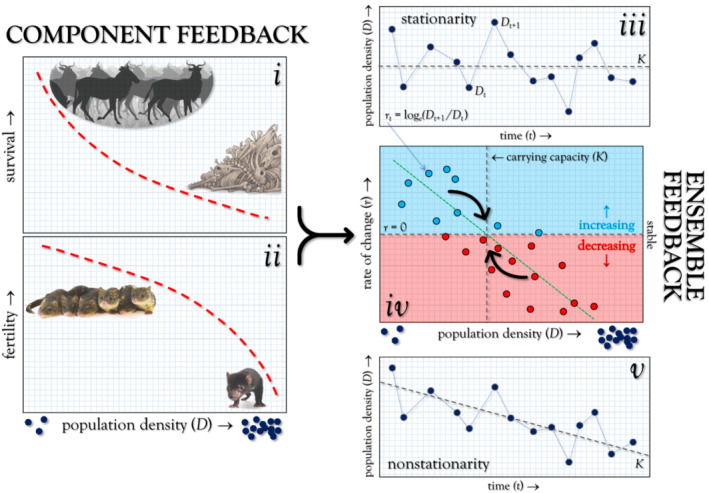
Scheme of the main elements of how density feedback operates in population dynamics (see Table 1 for a full glossary of terms indicated in italicized boldface). (*i*) a ***component density feedback*** can operate on survival probability (shown here as ***compensation***) where survival declines as population size increases. (*ii*) Another common component density feedback operates on fertility, where the number of offspring per female decreases with increasing population size. (*iii*) A time series of abundance estimates (“census data”) for a population captures an ***ensemble*** ***density feedback*** on the ***per capita*** ***rate of exponential population change*** (*r*) resulting from all component density feedbacks. In systems demonstrating ***stationarity***, the underlying mechanisms (e.g., ***carrying capacity** K*) driving change in population size do not themselves shift over time. (*iv*) Plotting the rate of population change (*r* = *N*_t + 1_/*N*_t_) against population size (*N*_t_) provides a way to measure the evidence for, and *strength* of, ensemble density feedback. In this representation, a ***Ricker logistic*** model estimates the linear slope between *r* and *N*_t_ (a negative slope here indicates ***compensation***, but a positive slope would indicate ***depensation***). Where *r* = 0 intersects the linear ***Ricker logistic*** fit, the long‐term mean ***carrying capacity*** (*K*) can be estimated if not trending upward or downward. The black arrows indicate that, under ***compensatory*** dynamics, a population tends to grow towards *K* when *r* > 0 (i.e., low *N*) and to decline from *K* when *r* < 0 (i.e., high *N*). (*v*) In this example, the system is in a state of ***nonstationarity*** because the *K* is declining over time.

Our overarching aim was to simulate populations of long‐lived species and their time series of abundance with known component feedback in survival (as well as in fertility in some cases to assess whether two component feedbacks altered our conclusions—they do not; see Appendix S1) and various sources of nonstationarity. Below, we describe the set of test species (Section 2.1), the simulation of the base population models for each species (Section 2.2) and of the component density feedbacks on survival within the base population models (Section 2.3), how we imposed (Section 2.4) and measured (Section 2.5) nonstationarity in the resultant time series of population abundance, how we simulated the abundance time series from the base population models (Section 2.6), the demographic scenarios we considered to examine which conditions led to a decoupling of component and ensemble feedbacks (Section 2.7), the logistic models we used to quantify ensemble density feedbacks from the projected time series of abundance (Section 2.8), and how we compared the strength of the component and ensemble density‐feedback signals (Section 2.9). See Figure 1 for a detailed schematic of the process.

### 2.1 Test species

Because the variability in population growth rates is driven primarily by variation in survival rates for species with slower life histories such as mammals (Oli & Dobson, 2003) and birds (Sæther & Bakke, 2000), we parameterised the simulated population dynamics of 21 long‐lived species of extant (*n* = 8) and extinct (*n* = 13) Australian vertebrates from five taxonomic/functional groups (herbivore vombatiformes [wombat suborder] and macropodiformes [kangaroo suborder], large omnivore birds, carnivores, and invertivore monotremes [echidnas]), spanning mean adult body masses of 1.7–2786 kg and generation lengths of 2.3–21 years (Bradshaw et al., 2021; Table 2). These species differ in their resilience to environmental change, and represent the slow end of the slow‐fast continuum of life histories (Herrando‐Pérez et al., 2012c). Here, high survival rates make it possible that reproductive efforts are dispersed over the lifetime of individuals (Gaillard et al., 1989). We chose this suite of species to cover a range of demographic types—it is the relative structure of the population and the particulars of the life histories that matter here, not the specifics of species A or B, or whether they are extant or extinct or live(d) in Australia or elsewhere. A full justification of the selection of our test species can be found in Bradshaw et al. (2021).

**TABLE 2:** Taxonomy and life‐history characteristics of the 21 test species (all native to Australia) used to simulate age‐structured populations and time series of population abundance (Bradshaw et al., 2021).

Taxonomic/functional group	Species	Abb	M	GL	*q*	Status
Herbivore vombatiformes	*Diprotodon optatum*	DP	2786	18.1	724	Extinct
	*Palorchestes azael*	PA	1000	15.1	604	Extinct
	*Zygomaturus trilobus*	ZT	500	13.2	528	Extinct
	*Phascolonus gigas*	PH	200	10.7	428	Extinct
	*Vombatus ursinus*	VU	25	10.0	400	Extant
Herbivore macropodiformes	*Procoptodon goliah*	PG	250	8.3	332	Extinct
	*Sthenurus stirlingi*	SS	150	8.1	324	Extinct
	*Protemnodon anak*	PT	130	7.8	312	Extinct
	*Simosthenurus occidentalis*	SO	120	7.8	312	Extinct
	*Metasthenurus newtonae*	MN	55	6.0	240	Extinct
	*Osphranter rufus*	OR	25	5.5	220	Extant
	*Notamacropus rufogriseus*	NR	14	6.3	252	Extant
Omnivore birds	*Genyornis newtoni*	GN	200	20.0	800	Extinct
	*Dromaius novaehollandiae*	DN	55	5.9	236	Extant
	*Alectura lathami*	AL	2.2	6.8	272	Extant
Carnivores	*Thylacoleo carnifex*	TC	110	9.1	364	Extinct
	*Thylacinus cynocephalus*	TH	20	5.2	208	Extinct
	*Sarcophilus harrisii*	SH	6.1	3.1	124	Extant^a^
	*Dasyurus maculatus*	DM	2	2.3	92	Extant
Invertivore monotremes	*Megalibgwilia ramsayi*	MR	11	16.4	656	Extant
	*Tachyglossus aculeatus*	TA	4	14.1	564	Extant

Abbreviations: GL, generation length (years); M, body mass (kg), *q*, projection length (years) of simulated populations given the species' GL.

^a^ Extant in Tasmania, currently extinct in mainland Australia.

### 2.2 Base (age‐structured) population model

We developed the base population model for each test species as a stochastic (i.e., parameters resampled within their uncertainty bounds) Leslie transition matrix (**M**) following a prebreeding design (Caswell, 2001; Table 1). The Leslie transition matrix **M** has *ω* + 1 (*i*) × *ω* + 1 (*j*) elements (ages from 0 to *ω* years) for females only, where *ω* = maximum longevity. Fertility (*m*_*x*_) occupied the first row of the matrix, survival probabilities (*S*_*x*_) occupied the subdiagonal, and the final diagonal transition probability (**M**_*i,j*_) was *S*_*ω*_ for all species―except *Vombatus ursinus* (VU; common wombat), *Thylacinus cynocephalus* (TC; thylacine), and *Sarcophilus harrisii* (SH; devil), for which we set *S*_*ω*_ = 0 to limit unrealistically high proportions of old individuals in the population given the evidence for catastrophic mortality at *ω* for the latter two species (Cockburn, 1997; Holz & Little, 1995; Oakwood et al., 2001). Multiplying **M** by a population vector **n** estimates total population size (Σ**n**) at each forecasted time step (Caswell, 2001). We parameterised the base model with **n**_0_ = *AD* **M**w for a closed population (dispersal = 0), where w is the right eigenvector of **M** (stable stage distribution), and *A* is the surface area of the study zone (*A* = 250,000 km^2^), so that the species with the lowest **n**_0_ would have an initial population of at least several thousand individuals at the start of the simulations. Based on theoretical equilibrium densities (*D*, km^−2^) calculated for each taxon (Bradshaw et al., 2021), we set the species‐specific carrying capacity *K* = *DA*.

We ran projections of the base model to 40 generations ($40\left\lfloor \left.G\right\rceil \right.$; see Section 2.6) per simulated population such that:

(1) $$G=\frac{\log\left({\left(v^{T}M\right)}_{1}\right)}{\lambda_{1}}$$

where (v^T^ **M**)_1_ is the dominant eigenvalue of the reproductive matrix **R** derived from **M**, and v is the left eigenvector of **M** (Caswell, 2001).

### 2.3 Component density feedback on survival

#### 2.3.1 Setting the survival modifier

We simulated a component compensatory density‐feedback function by forcing a reduction modifier (*S*_red_) of the age‐specific survival (*S*_*x*_) vector in **M** according to total population size Σ**n**:

(2) $$S_{\mathrm{red}}=\frac{a}{1+{\left(\frac{\sum n}{b}\right)}^{c}}$$

where the ***a***, ***b***, and ***c*** constants for each species are adjusted to produce a stable population on average over 40 generations ($40\left\lfloor \left.G\right\rceil \right.$; see above) (Brook et al., 2006; Traill et al., 2010). This formulation avoids exponentially increasing populations and optimizes transition matrices to produce parameter values as close as possible to the maximum potential rates of population increase (*r*_m_), therefore ensuring that long‐term population dynamics are approximately stable at the species‐specific carrying capacity.

The total projection length in years (*q*) varied across the 21 test species given their different generation lengths, from 92 (*Dasyurus maculatus*; DM; spot‐tailed quoll) to 800 (*Genyornis newtoni*; GN; mihirung) years (median = 324 years, with 95% interquartiles of 108–762 years; Table 2), with one value of abundance simulated per year representing a typical census interval (Knape & de Valpine, 2012).

#### 2.3.2 Varying uncertainty in survival

In each projection and annual time step, the survival vector *S*_*x*_ was resampled following a *β* distribution assuming a 5% standard deviation of each *S*_*x*_ and a Gaussian‐resampled fertility vector *m*_*x*_. We tested that increasing the standard deviation on juvenile survival (Barraquand & Yoccoz, 2013; Hilde et al., 2020) had no effect on our conclusions (see Appendix 2 and Section 3).

#### 2.3.3 Catastrophe function

For each species, we added a catastrophic (density‐independent) mortality function to the transition matrix **M** and scaled it to generation length among vertebrates (Reed et al., 2003):

(3) $$C=\frac{p_{C}}{G}$$

where ***p***_***C***_ = probability of catastrophe was set at 0.14 given this is the mean probability per generation observed across vertebrates (Reed et al., 2003). Once invoked at probability ***C***, a binomial *β*‐resampled proportion centred on 0.5 to the *β*‐resampled survival vector induces a ~ 50% mortality event for that year (Bradshaw et al., 2013). A catastrophic event is defined as “… any 1‐yr peak‐to‐trough decline in estimated numbers of 50% or greater” (Reed et al., 2003). The catastrophe function essentially recreates the demographic effects of a density‐independent process such as extreme weather events, fires, or disease outbreaks.

#### 2.3.4 Adding a component feedback in fertility

We deliberately avoided applying density‐feedback functions to fertility to isolate the component feedback to a single demographic rate (survival, see above). However, we also tested whether splitting the compensatory feedback between survival and fertility altered our results and conclusions (see Appendix 3 for justification and test outcomes). Our conclusions remained the same without or with a density feedback on fertility (Section 3).

### 2.4 Generating nonstationarity

Nonstationarity is defined as a property of a long‐term population trend whereby the density‐dependent and ‐independent mechanisms generating fluctuations in population density themselves change through time (Turchin, 2003; Table 1). To determine how nonstationarity affects the relationship between component and ensemble density feedbacks, we considered five main types of nonstationarity embedded within eight different demographic scenarios (see *Demographic scenarios* Section 2.7), as follows: (*i*) catastrophe survival function as the only source of nonstationarity (Section 2.3.3); (*ii*) catastrophe survival function with the addition of a 90% mortality pulse at 20 generations; (*iii*) increased mortality via a proportional offtake in the abundance vector (**n**) such that the population declined on average over the projection interval (two rates of population decline considered); (*iv*) variable but declining carrying capacity; (*v*) catastrophe survival function increased to produce a stable long‐term population trend ($\bar{r}$ ≅ 0) over 40 generations with a null density feedback on survival. These nonstationary mechanisms recreate real situations experienced by wild populations of large‐bodied carnivores and herbivores exposed to temporal changes in food resources or mortality events resulting from disease outbreaks or harvesting.

### 2.5 Measuring nonstationarity in abundance time series

To ascertain the degree of nonstationary in each simulated abundance time series (Section 2.6) across all demographic scenarios (Section 2.7), we calculated the mean and variance of return time (*T*_R_)—defined as the time required to return to equilibrium following a disturbance (Berryman, 1999). We calculated the mean and variance of return time for each generated abundance time series as:

(4) $${\bar{T}}_{R}=\frac{\sum_{m=1}^{M}T_{R_{m}}}{M}$$

where ${\bar{T}}_{R}$ is the mean *T*_R_ across ***M*** steps of the time series. For each ***m*** ^th^ time step,

(5) $$T_{R_{m}}=S_{C_{m}}+S_{F_{m}}$$

where $S_{C_{m}}$ is the number of complete time steps taken before reaching $T_{R_{m}}$, and $S_{F_{m}}$ is the fraction of time required to reach $T_{R_{m}}$ in the ***M*** ^th^ (final) step:

(6) $$S_{F_{m}}=\frac{N_{p}-\bar{N}}{N_{p}-N_{a}}$$

where $\bar{N}$ is the abundance mean across all time steps in the time series (a proxy for carrying capacity), ***N***_***p***_ is the population size prior to crossing $\bar{N}$, and ***N***_***a***_ is the population size after crossing $\bar{N}$. The variance of ***T***_**R**_ is:

(7) $$\mathrm{Var}\left(T_{R}\right)=\frac{\sum_{m=1}^{M}{\left(T_{R_{m}}-{\bar{T}}_{R}\right)}^{2}}{M-1}$$

Thus, when ${\bar{T}}_{R}\ll \mathrm{Var}\left(T_{R}\right)$ (i.e., ${\bar{T}}_{R}/\mathrm{Var}\left(T_{R}\right)\ll 1$), the time series is considered to be highly nonstationary (Berryman, 1999). See Appendix 1 and Figures S1–S3 for how these the perturbations imposed in the demographic scenarios altered indices of nonstationarity.

### 2.6 Simulating time series of population abundance

From the base model **M** that incorporates age structure, density feedbacks on survival, catastrophic events, and varying carrying capacity as described above, we generated multiannual abundance time series up to 40 generations for each species (Section 2.2; Equation 1). We standardized projection length to 40 generations because there is strong evidence that the length of a time series (*q*) dictates the statistical power to detect an ensemble density‐feedback signal in logistic growth curves (Brook & Bradshaw, 2006; Knape & de Valpine, 2012). Here, we summed the **n** abundance vector over all age classes to produce a total population size *N*_*t*,*i*_ for each year *t* of each projection *i*. We rejected the first generation of each projection as a burn‐in to allow the initial (deterministic) age distribution to calibrate to the stochastic expression of stability under compensatory density feedback.

### 2.7 Demographic scenarios

We generated 10,000 abundance time series over 40 generations (Sections 2.2 and 2.6) for each of the 21 test species (Table 2) in each of nine demographic scenarios (totalling 10,000 × 21 × 9 = 189,000 time series; 90,000 time series per species; 21,000 time series per scenario). Each times series represented the idiosyncratic demography of a unique population occupying an area of 250,000 km^2^ with zero permanent dispersal (Section 2.2).

Below, we present the nine demographic scenarios (summarized in Table 3), and then we describe the measurement of ensemble and compensatory feedbacks (*statistical support* in Section 2.8 and *strength* in Section 2.9) from each simulated time series across scenarios. Our set of scenarios emulate true nonstationary processes (Section 2.4; Appendix 1) often shaping the long‐term population dynamics of large mammals through density‐independent (catastrophic and harvest) mortality and variation in carrying capacity. Our focus is on whether those processes erode the density‐feedback signal from time series of abundance and precipitate decoupling of component and ensemble density feedbacks. Scenarios *i* to *viii* address the effects of nonstationary processes on ensemble density feedbacks when a component density feedback on survival is present (true positive), and Scenario *ix* addresses those effects when such a component feedback is absent, potentially leading to spurious ensemble density feedback (false positive).

**TABLE 3:** Demographic scenarios to quantify the detection of ensemble density‐feedback signals in time series of abundance using phenomenological models (logistic growth curves) if a component density feedback on survival is present (1. H_0_: false negatives), or absent (2. H_0_: false positives).

Scenario	Catastrophe type	Description
Component feedback present		
*Stochastic mortality*, *no catastrophic mortality*, *stable K*		
*K*_fixed_, $\bar{r}$ ≅ 0	none	Stochastically resampled survival rates in age‐structured population
*Catastrophic mortality (50%)*, *stable K*		
ii *K*_fixed_; $\bar{r}$ ≅ 0; sustained catastrophic mortality	generationally scaled	As in *i*, but with catastrophes
iii *K*_fixed_; $\bar{r}$ ≅ 0; additional pulsed catastrophic mortality	generationally scaled	As in *ii*, but with a single 90% mortality pulse implemented at 20*G*
*Harvest mortality*, *catastrophic mortality*, *stable K*		
iv *K*_fixed_; $\bar{r}$ ≅ −0.001; annual harvesting	generationally scaled	As in *ii*, but with proportional removal of individuals from the **n** vector such that $\bar{r}$ = −0.001 (slowly declining *N*)
v *K*_fixed_; $\bar{r}$ ≅ −0.01; annual harvesting	generationally scaled	As in *iv*, but where $\bar{r}$ = −0.01 (rapidly declining *N*)
vi *K*_stochastic_; $\bar{r}$ ≅ 0	generationally scaled	As in *ii*, but normally distributed *K* varying randomly at each time step (SD = 5%)
vii *K*_stochastic_ with increasing variance; $\bar{r}$ ≅ 0	generationally scaled	As in *vi*, but variance in *K* increased linearly from 5% to 10%
viii *K*_stochastic_ declining, forcing $\bar{r}$ < 0	generationally scaled	As in *vi*, but *K* also decreases on average at a rate of −0.001
Component feedback absent		
ix no *K*; $\bar{r}$ ≅ 0	temporally scaled	Probability of catastrophe increased over time such that $\bar{r}$ ≅ 0 (~ average stability)

*Note*: All scenarios were simulated over 40 generations across 21 test species (Table 2). Time series obtained from simulated age‐structured populations (Leslie matrices) occupying 250,000 km^2^ with no permanent dispersal.

Abbreviations: *G*, generation; *N*, population abundance; *K*, carrying capacity; $\bar{r}$, long‐term mean instantaneous rate of population change, SD, standard deviation.

#### 2.7.1 Stochasticity in demographic rates (Scenario *i*)

Scenario *i*: Population subjected to the stochasticity imposed by resampling demographic rates in the Leslie matrices (Section 2.2) (Dennis et al., 2006). This is the only scenario where we impose no catastrophic mortality events.

#### 2.7.2 Catastrophic mortality (scenarios *ii* and *iii*)

Scenario *ii*: As in Scenario *i*, but with generationally scaled catastrophes centered on 50% mortality, leading to population stability ($\bar{r}$ ≅ 0). Compared to Scenario *i*, Scenario *ii* tests the hypothesis that density‐independent catastrophes imposing process error erode the density‐feedback signal from time series of abundance (Abadi et al., 2012; Knape & de Valpine, 2012).

Scenario *iii*: As in Scenario *ii*, but with an additional, single “pulse” perturbation of 90% mortality applied across all ages at 20 generations to alter the population age structure—this tests the hypothesis that large “resets” of population size modify the underlying component dynamics so abruptly via highly modified age structure that the ensemble signal is eroded (Hoy et al., 2020;Turchin, 2003 ; Wu et al., 2007).

#### 2.7.3 Harvest‐like mortality (scenarios *iv* and *v*)

Scenario *iv*: A “harvest”‐like scenario where a consistent proportion of individuals is removed from the **n** abundance vector at each time step to produce a weakly declining population on average ($\bar{r}$ ≅ −0.001) (Bargmann et al., 2020; Bergman et al., 2015) (this scenario also includes the castastrophic mortality function described in Scenario *ii*).

Scenario *v*: As in Scenario *iv*, but with a strongly declining population on average ($\bar{r}$ ≅ −0.01). Scenarios *iv* and *v* test the hypothesis that the greater the rate of trending in population size over time, the more the ensemble signal is degraded.

#### 2.7.4 Variable carrying capacity (scenarios *vi*, *viii*, *viii*)

Scenario *vi*: Resampling a constant mean carrying capacity (and constant variance via resampling the *b* parameter in Equation 2). This tests the hypothesis that uncertainty in carrying capacity reduces ensemble feedbacks in abundance time series (Abadi et al., 2012; Knape & de Valpine, 2012). This scenario also includes the catastrophic mortality function described in Scenario *ii*.

Scenario *vii*: As in Scenario *vi*, but where the resampling variance in carrying capacity doubles over the projection interval (via a linear increase in the standard error used to resample the *b* parameter in Equation 2) (Abadi et al., 2012; Knape & de Valpine, 2012).

Scenario *viii*: As in Scenario *vi*, but with declines in carrying capacity at a rate of 0.001 over the projection interval (via decreasing the *b* parameter in Equation 2). This tests the hypothesis that state shifts (here, gradually reducing carrying capacity) erode the ensemble signal (Turchin, 2003; Wu et al., 2007).

#### 2.7.5 Absence of component density feedback on survival (Scenario *ix*)

Scenario *ix*: This is the only scenario where we imposed no component density feedback on survival, testing the hypothesis that in populations exposed to high density‐independent process error, false detection of an ensemble signal can occur even when component feedback is weak or absent (Knape, 2008). To produce populations that were approximately stable on average over the entire projection interval, we simulated density‐independent mortality via an increase in the probability of a catastrophe (*p*_*C*_ in Equation 3) to produce a stable population on average ($\bar{r}$ ≅ 0) over 40 generations, and removed the component density‐feedback on survival by setting the survival reduction parameter *S*_red_ to 1 in all iterations.

### 2.8 Measuring ensemble density feedbacks

For each simulated time series, we applied four phenomenological models to quantify both the statistical *evidence* of the ensemble compensatory density feedback and the *strength* of such a feedback as follows:

#### 2.8.1 Phenomenological models

The phenomenological models included four variants of the general logistic growth curve (Verhulst, 1838) following Brook and Bradshaw (2006):

(8) $$r=\log_{e}\left(\frac{N_{t+1}}{N_{t}}\right)=\alpha +\beta N_{t}+\varepsilon_{t}$$

where ***N***_***t***_ = population size at time ***t***, ***α*** = intercept, ***β*** = strength of ensemble density feedback, and ***ε***_***t***_ = Gaussian random variable with a mean of zero and a variance *σ* ^2^ reflecting uncorrelated stochastic variability in the per‐capita rate of population change ***r***. Our first two models are density‐independent models assuming no compensatory ensemble density feedback (DI): (1) random walk, where ***α*** = ***β*** = 0, and (2) exponential growth where ***β*** = 0. The second two variants are density‐feedback (or density‐dependent) models assuming a compensatory ensemble density feedback (DF): (3) Ricker‐logistic (Ricker, 1954), and (4) Gompertz‐logistic (Nelder, 1961), where ***N***_***t***_ on the right side of Equation 8 is replaced with **log**_***e***_(***N***_***t***_). The latter two models represent alternative situations where population growth rate varies in response to unit (Ricker) or order‐of‐magnitude (Gompertz) changes in population density (Herrando‐Pérez et al., 2012b).

#### 2.8.2 Strength of ensemble density feedback

We estimated the *strength* of the ensemble density‐feedback as the negative of the slope $\hat{\beta }$ estimated from the Gompertz‐logistic model (under compensation, $\hat{\beta }$ will always be < 0, so the lower the $\hat{\beta }$, the stronger the compensatory feedback). We used the Gompertz‐logistic $\hat{\beta }$, instead of the Ricker‐logistic $\hat{\beta }$, to estimate this strength because only the former characterizes the multiplicative nature of demographic rates (Doncaster, 2008; Herrando‐Pérez et al., 2012a).

#### 2.8.3 Statistical evidence for ensemble density feedback

We calculated the relative likelihood of the four phenomenological models fitted to each time series by means of the Akaike's information criterion (AIC) corrected for finite number of samples (AIC_*c*_) (Sugiura, 1978) in a multimodel inferential framework (Burnham & Anderson, 2002). Across the four models, we ranked the statistical *evidence* for an ensemble density‐feedback Pr(*density feedback*) as the sum of AIC_*c*_ weights (*w*AIC_*c*_ = model probability) for the Ricker‐ and Gompertz‐logistic models (i.e., Σ*w*AIC_*c*_‐*density feedback*), and the *evidence* for a lack of such feedback as the sum of AIC_*c*_ weights for random walk and exponential growth (i.e., Σ*w*AIC_*c*_‐*density independence*)—where Σ*w*AIC_*c*_‐*density feedback* + Σ*w*AIC_*c*_‐*density independence* = 1 (Burnham & Anderson, 2002). This follows the logic that the more the slope between the per‐capita rate of change (*r*) and abundance (*N*_*t*_) (Ricker model) *or* log_*e*_(*N*_*t*_) (Gompertz model) differs from zero (*β* ≠ 0), the stronger statistical support for an ensemble density feedback in the time series than density independence (Σ*w*AIC_*c*_‐*density feedback* > Σ*w*AIC_*c*_‐*density independence* implies Pr(*density feedback*) > 0.5)—providing that sample size (number of transitions) does not limit statistical inference (Herrando‐Pérez et al., 2012c).

### 2.9 Correlating ensemble versus component density feedbacks

We plotted the estimated strength of the ensemble density feedback (Gompertz‐$\hat{\beta }$) to the strength of the component feedback signal for survival (1 – *S*_red_) across all 21 species (Table 2) to determine whether the component strength can be used to predict the ensemble strength in each of the nine demographic scenarios. We tested the correlation between the strength of ensemble and component density feedbacks, and between the strength of ensemble feedback and the degree of nonstationarity, across species by calculating a bootstrapped estimate of Spearman's correlation *ρ* (treating relative differences in the metrics as ranks). We uniformly resampled 10,000 times from the 95% confidence interval of each metric for each species and demographic scenario, calculating the correlation coefficient *ρ* in turn, and then calculating the median and 95% confidence interval of *ρ*. The relationship between the strength of ensemble and component density feedback (as well as between ensemble strength and stationarity) showed some nonlinearity, so we also fitted simple exponential plateau models of the form *y* = *y*_m–x_ − (*y*_m–x_ − *y*_0_)e^−*kx*^ to these relationships. Here, *y*_0_ is the starting value of component strength, *y*_max_ is the maximum component strength (Gompertz‐$\hat{\beta }$), *k* = rate constant (in units of *x* ^−1^), and *x* is the component strength (1 – *S*_red_).

## 3 RESULTS

### 3.1 Magnitude of ensemble density feedbacks

**FIGURE 2:**
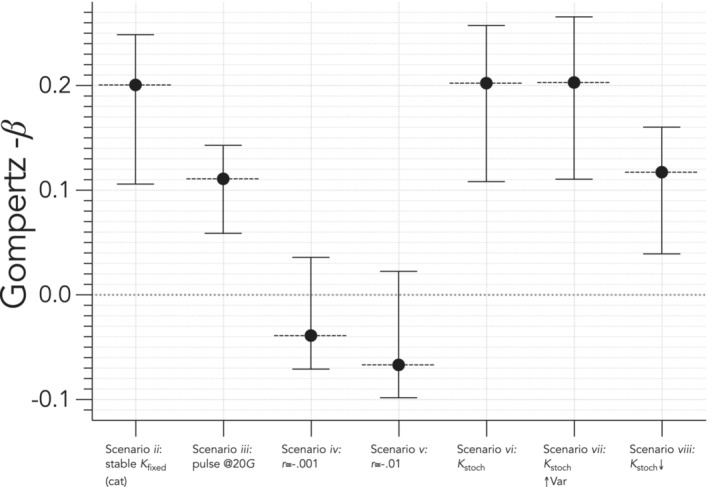
Strength of ensemble compensatory density feedback across demographic scenarios. Bootstrapped (10,000 uniform resamples between 95% confidence limits) across 21 test species (detailed in Table 2) of the strength of ensemble compensatory density feedback (Gompertz‐*β*) among scenarios (detailed in Table 3). Midpoints indicate means, and error bars are the interquartile ranges. Demographic scenarios include carrying capacity *K* fixed (*K*_fixed_; Scenario *ii*), a pulse disturbance of 90% mortality at 20 generations (20*G*; Scenario *iii*), weakly declining ($\bar{r}$ ≅ −0.001; Scenario *iv*) and strongly declining ($\bar{r}$ ≅ −0.01; Scenario *v*) populations, *K* varying stochastically (*K*_stoch_) around a constant mean with a constant variance (Scenario *vi*), *K* varying stochastically with a constant mean and increasing variance (*K*_stoch_↑Var; Scenario *vii*), and *K* varying stochastically with a declining mean and a constant variance (↓*K*_stoch_; Scenario *viii*).

Bootstrapping across all species, the reduction in ensemble density‐feedback strength measured as Gompertz‐*β* was greatest in Scenarios *iv* and *v* where we imposed population declines of $\bar{r}$ ≅ −0.001 and $\bar{r}$ ≅ −0.01, respectively, relative to the baseline Scenario *ii* ($\bar{r}$ ≅ 0) with population stability over time (Figure 2). The next largest reductions in the ensemble signal occurred in Scenarios *iii* (pulse perturbation at 20 generations) and *viii* (stochastically varying carrying capacity declining over time) (Figure 2). Lastly, Scenarios *vi* (stochastically varying carrying capacity around a long‐term stable average) and *viii* (stochastically varying carrying capacity around a long‐term stable average, with increasing variance over time) had similar ensemble feedback strengths relative to the base Scenario *ii* (Figure 2). Clearly, only harvest‐like mortality (Scenarios *iv* and *v*) dampens the strength of compensatory density feedbacks on population growth rates.

### 3.2 Strength of component versus ensemble density feedback

#### 3.2.1 Component‐ensemble decoupling

Decoupling of component and ensemble density feedback was signalled by the reduction in the correlation and/or the slope of the linear relationship between the strengths of both types of feedback for each time series across the 10,000 series covering 40 generations of each of the 21 test species and nine demographic scenarios. Neither increasing the standard deviation in juvenile survival relative to adults (Appendix 2; Figure S4), nor including a component feedback in fertility in addition to one operating on survival (Appendix 3), affected our conclusions.

**FIGURE 3:**
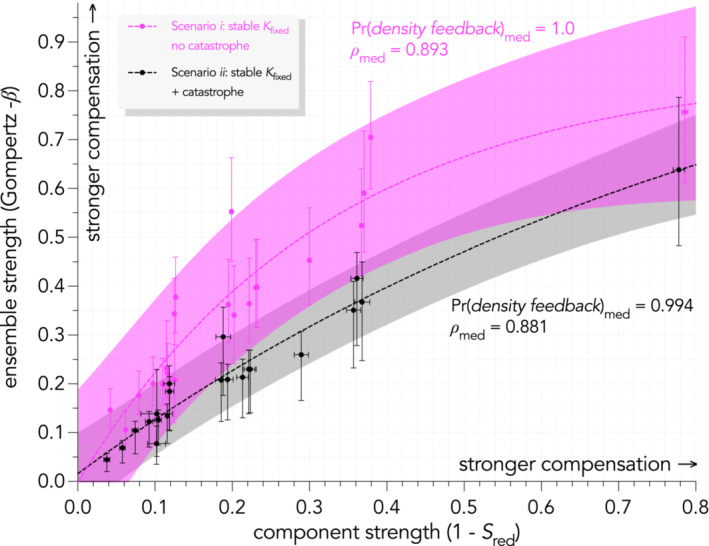
Decoupling of ensemble and component density feedbacks in demographic scenarios with and without catastrophic mortality. Relationship between strength of ensemble (slope coefficient *β* of the Gompertz‐logistic model × [−1] in the time series) and component (1 – the modifier *S*_red_ on survival in the Leslie transition matrix) density feedback for: Scenario *i* (pink; stochastic mortality, no catastrophic mortality, stable *K*) and Scenario *ii* (grey: stochastic mortality, catastrophic mortality, stable *K*). Fitted curves across species are exponential plateau models of the form *y* = *y*_max_ − (*y*_max_ − *y*_0_)e^−*kx*^. Shaded regions represent the 95% prediction intervals for each scenario. Each scenario includes 21,000 simulated time series of abundance (10,000 for each of 21  species; Table 2). Also shown are the mean probabilities of median density feedback (Pr(*density feedback*): sum of the Akaike's information criterion weights for the Ricker‐ and Gompertz‐logistic models across time series (Σ*w*AIC_*c*_‐*density feedback*) relative to the weights of two density‐independent models (random and exponential).

**FIGURE 4:**
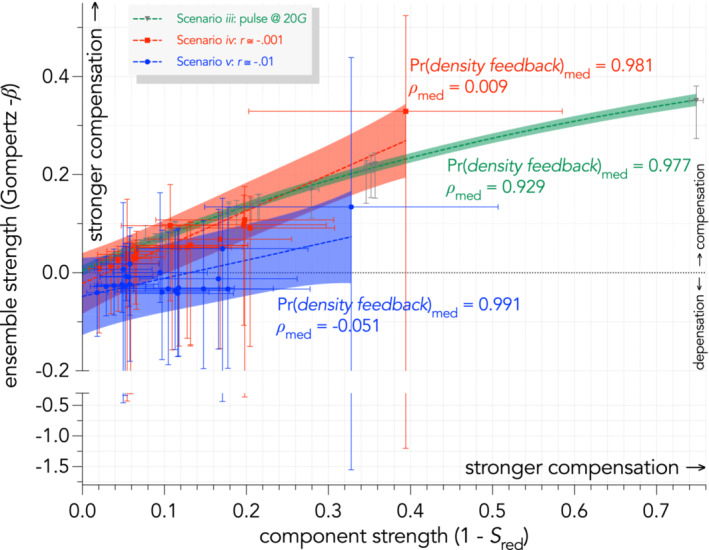
Decoupling of ensemble and component density feedbacks in demographic scenarios with catastrophic mortality and with catastrophic mortality + pulsed mortality and harvesting (see Figure 6). Relationship between strength of ensemble (slope coefficient *β* of the Gompertz‐logistic model × [−1]) and component (1 – the modifier *S*_red_ on survival) density feedback for: Scenario *iii* (green: pulse disturbance of 90% mortality at 20 generations); Scenario *iv* (red: weakly declining population at *r* ≅ −0.001); and Scenario *iv* (blue: strongly declining population at *r* ≅ −0.01). Each scenario includes 21,000 simulated time series of abundance (10,000 for each of 21 species; Table 2). Fitted curves across species are exponential plateau models of the form *y* = *y*_max_ − (*y*_max_ − *y*_0_)e^−*kx*^. Shaded regions represent the 95% prediction intervals for each scenario. Also shown are the mean probabilities of median density feedback (Pr(*density feedback*): sum of the Akaike's information criterion weights for the Ricker‐ and Gompertz‐logistic models across time series (Σ*w*AIC_*c*_‐*density feedback*) relative to the weights of two density‐independent models (random and exponential).

The addition of catastrophic mortality (Scenario *ii*) versus a population with only stochastic survival rates over the same period (Scenario *i*) reduced the correlation (median Spearman's *ρ* = 0.893 [0.826–0.947] and 0.881 [0.780–0.949], respectively) and slope between the strength of ensemble (Gompertz‐*β*; Section 2.8.2) and component feedback (1 – *S*_red_) across the 21,000 abundance time series (10,000 series × 21 test species) (Figure 3 and Figure S8). The catastrophic‐pulse mortality (Scenario *iii*) returned the closest correlation (median Spearman's *ρ* = 0.929 [0.871–0.971]) between the strength of ensemble and component feedback, although it also depressed the slope of the relationship relative to Scenario *i* (Figure 4).

**FIGURE 5:**
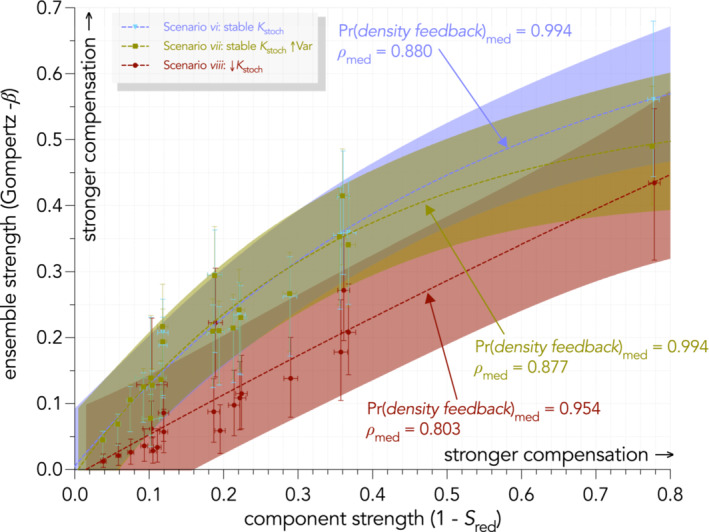
Decoupling of ensemble and component density feedbacks in demographic scenarios with catastrophic mortality and fluctuating carrying capacity. Relationship between strength of ensemble (slope coefficient *β* of the Gompertz‐logistic model × [−1]) and component (1 – the modifier *S*_red_ on survival) density feedback for: Scenario *vi* (purple: carrying capacity varying stochastically with a constant mean and an increasing variance); Scenario *vii* (green: carrying capacity varying stochastically with a constant mean and an increasing variance); and Scenario *viii* (red: carrying capacity *K* varying stochastically with a declining mean and a constant variance). Each scenario includes 21,000 simulated time series of abundance (10,000 for each of 21 test species, Table 2). Fitted curves across species are exponential plateau models of the form *y* = *y*_max_ − (*y*_max_ − *y*_0_)e^−*kx*^. Shaded regions represent the 95% prediction intervals for each scenario. Also shown are the mean probabilities of median density feedback (Pr(*density feedback*): sum of the Akaike's information criterion weights for the Ricker‐ and Gompertz‐logistic models across time series (Σ*w*AIC_*c*_‐*density feedback*) relative to the weights of two density‐independent models (random and exponential).

**FIGURE 6:**
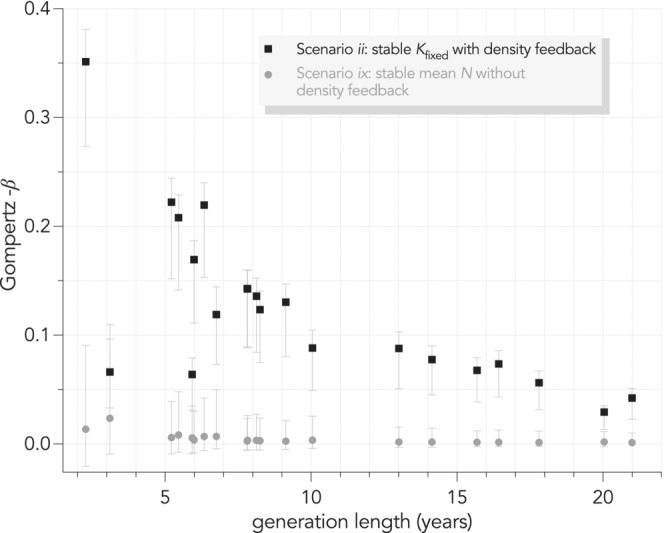
Strength of ensemble density feedback and generation length for 21 vertebrate species for demographic scenarios with and without a component density feedback on mortality. Relationship between strength of ensemble (slope coefficient *β* × [−1] of the Gompertz‐logistic model) and generation length across the 21 species for: Scenario *ii* (black: with compensatory density feedback; see also Figure 2) and Scenario *ix* (grey: without compensatory density feedback). Each scenario includes 21,000 simulated time series of abundance (10,000 for each of 21 test species, Table 2). Probabilities of density feedback (Pr(*density feedback*) = sum of the Akaike's information criterion weights for the Ricker and Gompertz models relative to the weights of two density‐independent models (random and exponential)) calculated across simulations gave median Pr(*density feedback*) = 0.994 and 0.322 for the two stable scenarios with (Scenario *ii*) and without (Scenario *ix*) component feedback on survival, respectively.

The magnitude of correlation when the carrying capacity was forced to fluctuate (Figure 5) ranged from a median Spearman's *ρ* of 0.8 to 0.9 for Scenarios *vi* to *viii* (Figure 5 and Figure S8). In contrast, strong decoupling occurred in the harvest‐mortality scenarios, with median Spearman's *ρ* of only 0.009 [−0.441–0.489] (Scenario *iv*) and −0.051 [−0.498–0.412] (Scenario *v*) (Figure 4). Noticeably, some abundance time series experienced depensation or “Allee effects” (population growth rate increasing with population size; Table 1). For these two harvest‐like scenarios (Scenarios *iv* and *v*), the 95% confidence interval of the ensemble component strength included 0 for all species (Figure 4). As expected, when the component density feedback on survival was absent (Scenario *ix*), all estimated strengths of ensemble feedback enveloped 0 (Figure 6), meaning an absence of an ensemble density‐feedback signal (i.e., *r ~* log_*e*_(*N*_*t*_) slope not differentiated from zero). Clearly, the decoupling between the density feedback on population growth rates (ensemble) and mortality (component) varied according to the type of perturbation the populations experienced, with the strongest decoupling caused by harvest‐like mortality.

#### 3.2.2 Strength of ensemble feedback versus nonstationarity

**FIGURE 7:**
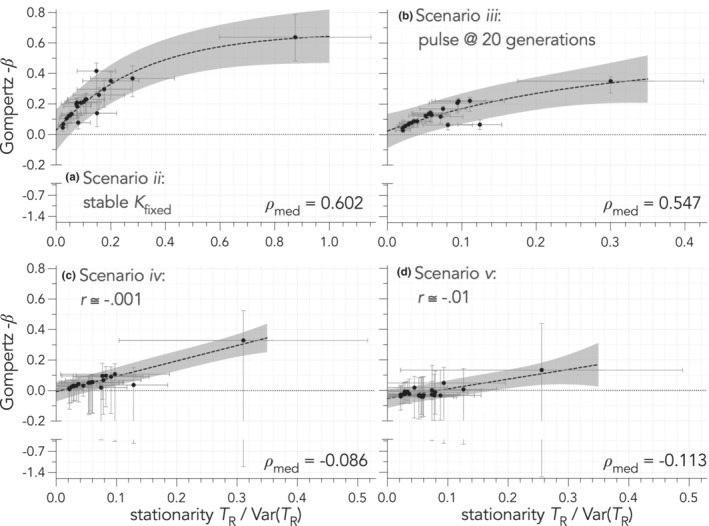
Strength of ensemble density feedback in demographic scenarios with catastrophic mortality, catastrophic mortality with pulsed mortality, and two types of harvesting. Relationship between strength of ensemble density feedback (slope coefficient *β* × [−1] of the Gompertz‐logistic model) and the stationarity index ${\bar{T}}_{R}/\mathrm{Var}\left(T_{R}\right)$ across 21 test species over 40 generations for four demographic scenarios: (a) Scenario *ii*: carrying capacity (*K*) fixed, (b) Scenario *iii*: a pulse disturbance of 90% mortality at 20 generations, (c) Scenario *iv*: weakly declining population at *r* ≅ −0.001, and (d) Scenario *v*: strongly declining population at *r* ≅ −0.01. Each scenario includes 21,000 simulated time series of abundance (10,000 for each of 21 species, Table 2). Fitted curves across species exponential plateau models of the form *y* = *y*_max_ − (*y*_max_ − *y*_0_)e^−*kx*^. Shaded regions represent the 95% prediction intervals for each type. *ρ*_med_ are the median Spearman's *ρ* correlation coefficients for the relationship between the ensemble strength and stationarity index across species (resampled 10,000 times; see Figure S8 for full uncertainty range of *ρ* in each scenario).

**FIGURE 8:**
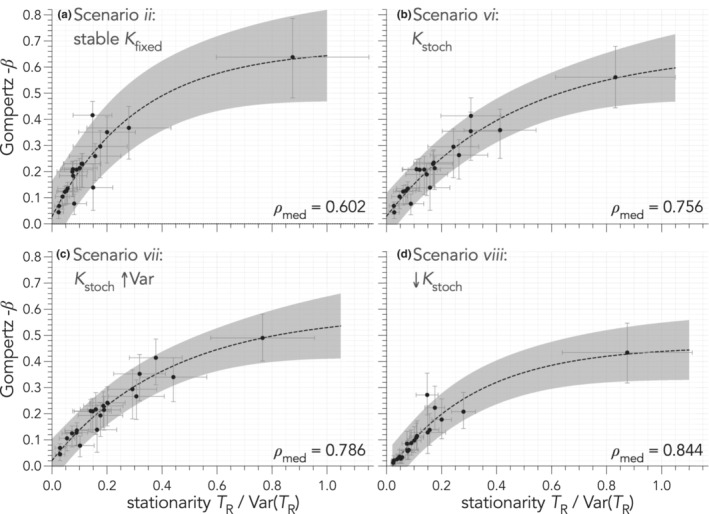
Strength of ensemble density feedback in demographic scenarios with catastrophic mortality and fixed carrying capacity versus three types of fluctuating carrying capacity and no catastrophic mortality. Relationship between strength of ensemble density feedback (slope coefficient *β* × [−1] of the Gompertz‐logistic model) and the stationarity index ${\bar{T}}_{R}/\mathrm{Var}\left(T_{R}\right)$ across 21 test species over 40 generations for four demographic scenarios: (a) Scenario *ii*: carrying capacity (*K*) fixed, (b) Scenario *vi*: *K* varying stochastically (*K*_stoch_) around a constant mean with a constant variance, (c) Scenario *vii*: *K* varying stochastically with a constant mean and increasing variance (*K*_stoch_ ↑Var), and (d) Scenario *viii*: *K* varying stochastically with a declining mean and a constant variance (↓*K*_stoch_). Each scenario includes 21,000 simulated time series of abundance (10,000 for each of 21 species. Fitted curves across species exponential plateau models of the form *y* = *y*_max_ − (*y*_max_ − *y*_0_)e^−*kx*^. Shaded regions represent the 95% prediction intervals for each type. *ρ*_med_ are the median Spearman's *ρ* correlation coefficients for the relationship between the ensemble strength and stationarity index across species (resampled 10,000 times; see Figure S8 for full uncertainty range under each scenario).

Nonstationarity was a weak (median Spearman's *ρ* = −0.113 – −0.086 over 10,000 time series × 21 test species) predictor of the strength of ensemble feedback when catastrophic (Scenarios *ii*, *iii*) or harvest‐like (Scenarios *iv*, *v*) mortality was imposed (Figure 7), but both variables were reasonably well‐correlated (median Spearman's *ρ* = 0.756–0.844) for Scenarios *vi* to *viii* with fluctuating carrying capacity (Figure 8 and Figure S8). The former correlations indirectly reinforce the observation that density‐independent mortality is a stronger driver of component‐ensemble density‐feedback decoupling than fluctuating resources (Subsection 3.2.1) as the variation in the magnitude of density feedbacks is more responsive to variation in carrying capacity than to density‐independent mortality.

### 3.3 Evidence for density feedback

The magnitude of statistical evidence for density feedback was largely invariant across all demographic scenarios (*i* to *viii*) that had a component feedback on survival (Figures S5 and S6; see above). Thus, the median probability for a signal of ensemble feedback (Pr(*density feedback*) = Σ*w*AIC_*c*_‐*density feedback* for Ricker and Gompertz models, see Section 2) over 21,000 abundance times series (10,000 series × 21 test species) was >0.99 for scenarios *i* to *vii* (Figures S5–S7), and 0.93 (0.74– > 0.99) for Scenario *viii* with a declining carrying capacity. Logically, for Scenario *ix* where we imposed a null density feedback on survival in our simulated time series, the median statistical support for an ensemble density feedback was only 0.32 (0.31–0.34), so the two models assuming no ensemble density feedback (random, exponential, see Section 2) received the highest statistical support. Finally, false positives in demographic Scenario *iv* (component feedback absent, ensemble feedback detected) occurred in <4 of every 10 time series.

In summary, if a component density feedback on survival was present (theoretically driving the ensemble density feedback on the population growth rate), the phenomenological models were reasonably good at *detecting* the ensemble feedback from the time series (true positive in >9 of every 10 time series)―regardless of whether a population was perturbed via fluctuating carrying capacity or catastrophic or harvest mortality .

## 4 DISCUSSION

Our simulations reveal several new insights into how density‐feedback signals in population growth rates and those operating on vital rates can be decoupled. First, we discovered that the estimated *strength* of density feedbacks from abundance time series are particularly sensitive to density‐independent mortality that produces long‐term declines in population size. In other words, logistic models are unlikely to reveal density feedback in harvested populations that are declining, even when strong component feedbacks exist. Therefore, attempting to measure density feedbacks in such populations only from time series of abundance would be unlikely to bear fruit. On the contrary, estimated feedback strength is much less sensitive to moderate fluctuations in carrying capacity.

Second, the statistical detection of density feedbacks in abundance time series is robust in the face of even pronounced nonstationarity. It is essential here to distinguish the *detection* from the *strength* of the feedback itself—the former is based on the statistical evidence that phenomenological models provide more support for a relationship between rate of change and population density than not (Brook & Bradshaw, 2006), whereas the latter indicates the magnitude of the slope of that relationship (Herrando‐Pérez et al., 2012c). Third, the concern that density‐independent processes can invoke false evidence of ensemble signals of compensation are not borne out by our simulations, at least with respect to density‐independent mortality not leading to declining population size. Our results therefore lend credence to the application of phenomenological (logistic‐growth) models to studies addressing the long‐term effect of vital rates on population abundance, provided there is enough information available (i.e., population censuses over long periods) for describing population trends.

The relative magnitude of density‐dependent and ‐independent mechanisms and their characterization and detection with logistic models will vary from population to population. For instance, variation in survival probability can be entirely driven by variation in climatic conditions and density‐independent predation (Hebblewhite et al., 2018). In one of the best‐studied systems in this regard, Soay sheep (*Ovis aries*) populations from St. Kilda Archipelago in the UK demonstrate that the demographic role of density and weather varies across sexes and age classes in mild winters, but survival is reduced consistently in all individuals in years of bad weather and when abundance is high (Coulson et al., 2001). An illustrative example with carnivores are wolves (*Canis lupus*) whereby interpack aggression with strong social hierarchies might shape survival at high densities, but become demographically irrelevant at low densities resulting from prey shortages and/or hunting or culling (Cubaynes et al., 2014). Our results reveal that such density‐independent processes can erode the ensemble signal if insufficient data are available relative to the frequency of such events.

Our approach and findings do not, of course, explain all possible scenarios leading to the decoupling of density‐feedback signals in single demographic rates and abundance time series. For example, other density‐independent factors that we did not consider can dampen the demographic role of social and trophic interactions mediated by population size (Herrando‐Pérez et al., 2012a), among the most important being immigration (Lieury et al., 2015) and spatial heterogeneity in population growth rates (Thorson et al., 2015). Indeed, examining the nuances of spatial heterogeneity and the exchange of individuals among populations would require a completely different modeling framework than the one we constructed here. Other disrupting phenomena such as fluctuating age structure (Hoy et al., 2020), environmental state shifts (Turchin, 2003; Wu et al., 2007), and sampling error (Knape & de Valpine, 2012) were implicit in our modeling framework. In addition, by standardizing the spatial extent and population densities at the beginning of all projections, and by including known sampling and process errors, our models quantify the contributions of nonstationarity and other forms of density‐independent change to vital rates.

Another caveat is that simulating closed populations might have potentially inflated our capacity to detect the component signal in abundance time series, because permanent dispersal could alleviate per‐capita reductions in fitness as a population nears carrying capacity. We also limited our projections to a standardized 40 generations across species, but even expanding these to 120 generations resulted in little change in the stationarity metric (Figure S9). Complementary studies focusing on the faster end of the life‐history continuum could provide further insights, even though our range of test species still produced a life‐history signal of the strength and stationarity of component (Figure S10) and ensemble density feedbacks (Figures S11 and S12) that declined with increasing generation length. However, this relationship faded when the trajectories simulated declines through proportional removal of individuals. Indeed, both evidence for (Holyoak & Baillie, 1996), and strength of (Herrando‐Pérez et al., 2012c), ensemble density feedback generally increase along the continuum of slow to fast life histories, because species with slow life histories are assumed to be more demographically stable when density compensation is operating (Sæther et al., 2002).

## 5 CONCLUSIONS

While quantifying the true extent of all component density‐feedback mechanisms operating in real populations will remain challenging in most circumstances, phenomenological models can normally capture the evidence for and strength of the component feedbacks at play. Appreciating the degree of nonstationarity and other types of perturbations affecting abundance time series can contextualize interpretations of estimated signals of density feedback in abundance time series, especially where substantial density‐independent mortality leads to long‐term population declines. Importantly, failing to capture the realistic magnitude of density‐feedback strength in applied ecological models can lead to suboptimal conservation and management recommendations and outcomes (Herrando‐Pérez et al., 2012a; Horswill et al., 2017).

## AUTHOR CONTRIBUTIONS

**Corey Bradshaw:** Conceptualization (lead); data curation (lead); formal analysis (lead); investigation (lead); methodology (lead); project administration (lead); resources (lead); software (lead); writing – original draft (lead); writing – review and editing (lead). **Salvador Herrando‐Pérez:** Methodology (supporting); writing – review and editing (supporting).

## FUNDING INFORMATION

Australian Research Council Centre of Excellence grant (CE170100015) to Corey J. A. Bradshaw. European Union's LIFE18 NAT/ES/000121 LIFE DIVAQUA to Salvador Herrando‐Pérez.

### OPEN RESEARCH BADGES

This article has earned Open Data and Open Materials badges. Data and materials are available at [https://github.com/cjabradshaw/DensityFeedbackSims].

## Supporting information


Appendix S1

## Data Availability

All data files and R code are openly available at https://github.com/cjabradshaw/DensityFeedbackSims.

## References

[ece310010-bib-0001] Abadi, F. , Gimenez, O. , Jakober, H. , Stauber, W. , Arlettaz, R. , & Schaub, M. (2012). Estimating the strength of density dependence in the presence of observation errors using integrated population models. Ecological Modelling, 242, 1–9. 10.1016/j.ecolmodel.2012.05.007

[ece310010-bib-0002] Bargmann, T. , Wheatcroft, E. , Imperio, S. , & Vetaas, O. R. (2020). Effects of weather and hunting on wild reindeer population dynamics in Hardangervidda National Park. Population Ecology, 62, 91–104. 10.1002/1438-390X.12030

[ece310010-bib-0003] Barraquand, F. , & Yoccoz, N. G. (2013). When can environmental variability benefit population growth? Counterintuitive effects of nonlinearities in vital rates. Theoretical Population Biology, 89, 1–11. 10.1016/j.tpb.2013.07.002 23906589

[ece310010-bib-0004] Battaile, B. C. , & Trites, A. W. (2013). Linking reproduction and survival can improve model estimates of vital rates derived from limited time‐series counts of pinnipeds and other species. PLoS One, 8, e77389. 10.1371/journal.pone.0077389 24324541 PMC3855591

[ece310010-bib-0005] Bergman, E. J. , Doherty, P. F., Jr. , White, G. C. , & Holland, A. A. (2015). Density dependence in mule deer: a review of evidence. Wildlife Biology, 21, wlb.00855. 10.2981/wlb.00012

[ece310010-bib-0006] Berryman, A. , & Turchin, P. (2001). Identifying the density‐dependent structure underlying ecological time series. Oikos, 92, 265–270. 10.1034/j.1600-0706.2001.920208.x

[ece310010-bib-0007] Berryman, A. A. (1989). The conceptual foundations of ecological dynamics. Bulletin of the Ecological Society of America, 70, 230–236.

[ece310010-bib-0008] Berryman, A. A. (1999). Principles of population dynamics and their application. Stanley Thorners Ltd.

[ece310010-bib-0009] Berryman, A. A. , Lima Arce, M. , & Hawkins, B. A. (2002). Population regulation, emergent properties, and a requiem for density dependence. Oikos, 99, 600–606. 10.1034/j.1600-0706.2002.12106.x

[ece310010-bib-0010] Bonebrake, T. C. , Christensen, J. , Boggs, C. L. , & Ehrlich, P. R. (2010). Population decline assessment, historical baselines, and conservation. Conservation Letters, 3, 371–378. 10.1111/j.1755-263X.2010.00139.x

[ece310010-bib-0011] Bonsall, M. B. , & Benmayor, R. (2005). Multiple infections alter density dependence in host‐pathogen interactions. Journal of Animal Ecology, 74, 937–945. 10.1111/j.1365-2656.2005.00991.x

[ece310010-bib-0012] Bradshaw, C. J. A. , Field, I. C. , McMahon, C. R. , Johnson, G. J. , Meekan, M. G. , & Buckworth, R. C. (2013). More analytical bite in estimating targets for shark harvest. Marine Ecology Progress Series, 488, 221–232. 10.3354/meps10375

[ece310010-bib-0013] Bradshaw, C. J. A. , Johnson, C. N. , Llewelyn, J. , Weisbecker, V. , Strona, G. , & Saltré, F. (2021). Relative demographic susceptibility does not explain the extinction chronology of Sahul's megafauna. eLife, 10, e63870. 10.7554/eLife.63870 33783356 PMC8043753

[ece310010-bib-0014] Brook, B. W. , & Bradshaw, C. J. A. (2006). Strength of evidence for density dependence in abundance time series of 1198 species. Ecology, 87, 1445–1451. 10.1111/j.1461-0248.2006.00883.x 16869419

[ece310010-bib-0015] Brook, B. W. , Traill, L. W. , & Bradshaw, C. J. A. (2006). Minimum viable population size and global extinction risk are unrelated. Ecology Letters, 9, 375–382. 10.1111/j.1461-0248.2006.00883.x 16623722

[ece310010-bib-0016] Bürgi, L. P. , Roltsch, W. J. , & Mills, N. J. (2015). Allee effects and population regulation: A test for biotic resistance against an invasive leafroller by resident parasitoids. Population Ecology, 57, 215–225. 10.1007/s10144-014-0451-4

[ece310010-bib-0017] Burnham, K. P. , & Anderson, D. R. (2002). Model selection and multimodel inference: a practical information‐theoretic approach (2nd ed.). Springer‐Verlag.

[ece310010-bib-0018] Caswell, H. (2001). Matrix population models: Construction, analysis, and interpretation (2nd ed.). Sinauer Associates, Inc.

[ece310010-bib-0019] Cockburn, A. (1997). Living slow and dying young: Senescence in marsupials. In N. Saunders & L. Hinds (Eds.), Marsupial biology: recent research, new perspectives (pp. 163–171). University of New South Wales Press.

[ece310010-bib-0020] Coulson, T. , Catchpole, E. A. , Albon, S. D. , Morgan, B. J. T. , Pemberton, J. M. , Clutton‐Brock, T. H. , Crawley, M. J. , & Grenfell, B. T. (2001). Age, sex, density, winter weather and population crashes in soay sheep. Science, 292, 1528–1531. 10.1126/science.292.5521.1528 11375487

[ece310010-bib-0021] Courchamp, F. , Clutton‐Brock, T. , & Grenfell, B. (1999). Inverse density dependence and the Allee effect. Trends in Ecology and Evolution, 14, 405–410. 10.1016/S0169-5347(99)01683-3 10481205

[ece310010-bib-0022] Cubaynes, S. , Macnulty, D. R. , Stahler, D. R. , Quimby, K. A. , Smith, D. W. , & Coulson, T. (2014). Density‐dependent intraspecific aggression regulates survival in northern Yellowstone wolves (*Canis lupus*). Journal of Animal Ecology, 83, 1344–1356. 10.1111/1365-2656.12238 24749694

[ece310010-bib-0023] Dennis, B. , Ponciano, J. M. , Lele, S. R. , Taper, M. L. , & Staples, D. F. (2006). Estimating density dependence, process noise, and observation error. Ecological Monographs, 76, 323–341. 10.1890/0012-9615(2006)76[323:EDDPNA]2.0.CO;2

[ece310010-bib-0024] Dennis, B. , & Taper, M. L. (1994). Density dependence in time series observations of natural populations: estimation and testing. Ecological Monographs, 64, 205–224. 10.2307/2937041

[ece310010-bib-0025] di Fonzo, M. M. I. , Collen, B. , Chauvenet, A. L. M. , & Mace, G. M. (2016). Patterns of mammalian population decline inform conservation action. Journal of Applied Ecology, 53, 1046–1054. 10.1111/1365-2664.12659

[ece310010-bib-0026] Doncaster, C. P. (2008). Non‐linear density dependence in time series is not evidence of non‐logistic growth. Theoretical Population Biology, 73, 483–489. 10.1016/j.tpb.2008.02.003 18395764

[ece310010-bib-0027] Eberhardt, L. L. (2002). A paradigm for population analysis of long‐lived vertebrates. Ecology, 83, 281–2854. 10.1890/0012-9658(2002)083[2841:APFPAO]2.0.CO;2

[ece310010-bib-0028] Eberhardt, L. L. , Breiwick, J. M. , & Demaster, D. P. (2008). Analyzing population growth curves. Oikos, 117, 1240–1246. 10.1111/j.0030-1299.2008.16402.x

[ece310010-bib-0029] Gaillard, J. M. , Pontier, D. , Allainé, D. , Lebreton, J. D. , Trouvilliez, J. , & Clobert, J. (1989). An analysis of demographic tactics in birds and mammals. Oikos, 56, 59–76. 10.2307/3566088

[ece310010-bib-0030] Gamelon, M. , Grøtan, V. , Engen, S. , Bjørkvoll, E. , Visser, M. E. , & Sæther, B. E. (2016). Density dependence in an age‐structured population of great tits: identifying the critical age classes. Ecology, 97, 2479–2490. 10.1002/ecy.1442 27859080

[ece310010-bib-0031] Gotelli, N. J. (2008). A primer of ecology. Sinauer Associates.

[ece310010-bib-0032] Hebblewhite, M. , Eacker, D. R. , Eggeman, S. , Bohm, H. , & Merrill, E. H. (2018). Density‐independent predation affects migrants and residents equally in a declining partially migratory elk population. Oikos, 127, 1304–1318. 10.1111/oik.05304

[ece310010-bib-0033] Herrando‐Pérez, S. , Delean, S. , Brook, B. W. , & Bradshaw, C. J. A. (2012a). Decoupling of component and ensemble density feedbacks in birds and mammals. Ecology, 93, 1728–1740. 10.1890/11-1415.1 22919918

[ece310010-bib-0034] Herrando‐Pérez, S. , Delean, S. , Brook, B. W. , & Bradshaw, C. J. A. (2012b). Density dependence: an ecological tower of babel. Oecologia, 170, 585–603. 10.1007/s00442-012-2347-3 22648068

[ece310010-bib-0035] Herrando‐Pérez, S. , Delean, S. , Brook, B. W. , & Bradshaw, C. J. A. (2012c). Strength of density feedback in census data increases from slow to fast life histories. Ecology and Evolution, 2, 1922–1934. 10.1002/ece3.298 22957193 PMC3433995

[ece310010-bib-0036] Hilde, C. H. , Gamelon, M. , Sæther, B.‐E. , Gaillard, J.‐M. , Yoccoz, N. G. , & Pélabon, C. (2020). The demographic buffering hypothesis: evidence and challenges. Trends in Ecology and Evolution, 35, 523–538. 10.1016/j.tree.2020.02.004 32396819

[ece310010-bib-0037] Holyoak, M. , & Baillie, S. R. (1996). Factors influencing detection of density dependence in British birds: II. Longevity and population variability. Oecologia, 108, 54–63. 10.1007/BF00333214 28307733

[ece310010-bib-0038] Holz, P. H. , & Little, P. B. (1995). Degenerative leukoencephalopathy and myelopathy in dasyurids. Journal of Wildlife Diseases, 31, 509–513. 10.7589/0090-3558-31.4.509 8592382

[ece310010-bib-0039] Horswill, C. , O'Brien, S. H. , & Robinson, R. A. (2017). Density dependence and marine bird populations: are wind farm assessments precautionary? Journal of Applied Ecology, 54, 1406–1414. 10.1111/1365-2664.12841

[ece310010-bib-0040] Hostetler, J. A. , & Chandler, R. B. (2015). Improved state‐space models for inference about spatial and temporal variation in abundance from count data. Ecology, 96, 1713–1723. 10.1890/14-1487.1

[ece310010-bib-0041] Hoy, S. R. , MacNulty, D. R. , Smith, D. W. , Stahler, D. R. , Lambin, X. , Peterson, R. O. , Ruprecht, J. S. , & Vucetich, J. A. (2020). Fluctuations in age structure and their variable influence on population growth. Functional Ecology, 34, 203–216. 10.1111/1365-2435.13431

[ece310010-bib-0042] Johnson, E. C. , Hastings, A. , & Ray, C. (2022). An explanation for unexpected population crashes in a constant environment. Ecology Letters, 25, 2573–2583. 10.1111/ele.14110 36317948

[ece310010-bib-0043] Knape, J. (2008). Estimability of density dependence in models of time series data. Ecology, 89, 2994–3000. 10.1890/08-0071.1 31766811

[ece310010-bib-0044] Knape, J. , & de Valpine, P. (2012). Are patterns of density dependence in the global population dynamics database driven by uncertainty about population abundance? Ecology Letters, 15, 17–23. 10.1111/j.1461-0248.2011.01702.x 22017744

[ece310010-bib-0045] Kolb, A. , Dahlgren, J. P. , & Ehrlén, J. (2010). Population size affects vital rates but not population growth rate of a perennial plant. Ecology, 91, 3210–3217. 10.1890/09-2207.1 21141182

[ece310010-bib-0046] Layton‐Matthews, K. , Loonen, M. J. J. E. , Hansen, B. B. , Coste, C. F. D. , Sæther, B.‐E. , & Grøtan, V. (2019). Density‐dependent population dynamics of a high Arctic capital breeder, the barnacle goose. Journal of Animal Ecology, 88, 1191–1201. 10.1111/1365-2656.13001 31032900

[ece310010-bib-0047] Levin, S. A. , Carpenter, S. R. , Godfray, H. C. J. , Kinzig, A. P. , Loreau, M. , Losos, J. B. , Walker, B. , & Wilcove, D. S. (Eds.). (2008). The Princeton guide to ecology. Princeton University Press.

[ece310010-bib-0048] Lieury, N. , Ruette, S. , Devillard, S. , Albaret, M. , Drouyer, F. , Baudoux, B. , & Millon, A. (2015). Compensatory immigration challenges predator control: an experimental evidence‐based approach improves management. Journal of Wildlife Management, 79, 425–434. 10.1002/jwmg.850

[ece310010-bib-0049] Ma, Z. (2021). A unified survival‐analysis approach to insect population development and survival times. Scientific Reports, 11, 8223. 10.1038/s41598-021-87264-1 33859237 PMC8050314

[ece310010-bib-0050] Marini, G. , Poletti, P. , Giacobini, M. , Pugliese, A. , Merler, S. , & Rosà, R. (2016). The role of climatic and density dependent factors in shaping mosquito population dynamics: the case of *Culex pipiens* in northwestern Italy. PLoS One, 11, e0154018. 10.1371/journal.pone.0154018 27105065 PMC4841511

[ece310010-bib-0051] Matthysen, E. (2005). Density‐dependent dispersal in birds and mammals. Ecography, 28, 403–416. 10.1111/j.0906-7590.2005.04073.x

[ece310010-bib-0052] McGeoch, M. A. , & Price, P. W. (2005). Scale‐dependent mechanisms in the population dynamics of an insect herbivore. Oecologia, 144, 278–288. 10.1007/s00442-005-0073-9 15891834

[ece310010-bib-0053] Medawar, P. B. (1940). The growth, growth energy, and ageing of the chicken's heart. Proceedings of the Royal Society B: Biological Sciences, 129, 332–355. 10.1098/rspb.1940.0042

[ece310010-bib-0054] Micheli, F. , Carlton, J. , Pearse, J. , Selgrath, J. , Elahi, R. , Watanabe, J. , Mach, M. , McDevitt‐Irwin, J. , Pearse, V. , Burnett, N. , & Baxter, C. (2020). Field stations as sentinels of change. Frontiers in Ecology and the Environment, 18, 320–322. 10.1002/fee.2231

[ece310010-bib-0055] Morrison, C. A. , Butler, S. J. , Robinson, R. A. , Clark, J. A. , Arizaga, J. , Aunins, A. , Baltà, O. , Cepák, J. , Chodkiewicz, T. , Escandell, V. , Foppen, R. P. B. , Gregory, R. D. , Husby, M. , Jiguet, F. , Kålås, J. A. , Lehikoinen, A. , Lindström, Å. , Moshøj, C. M. , Nagy, K. , … Gill, J. A. (2021). Covariation in population trends and demography reveals targets for conservation action. Proceedings of the Royal Society B: Biological Sciences, 288, 20202955. 10.1098/rspb.2020.2955 PMC7934962 33653129

[ece310010-bib-0056] Münster‐Swendsen, M. , & Berryman, A. (2005). Detecting the causes of population cycles by analysis of R‐functions: the spruce needle‐miner, *Epinotia tedella*, and its parasitoids in Danish spruce plantations. Oikos, 108, 495–502. 10.1111/j.0030-1299.2005.13747.x

[ece310010-bib-0057] Neave, F. (1953). Principles affecting the size of pink and chum salmon populations in British Columbia. Journal of the Fisheries Research Board of Canada, 9, 450–491.

[ece310010-bib-0058] Nelder, J. A. (1961). The fitting of a generalization of the logistic curve. Biometrics, 17, 89–110. 10.2307/2527498

[ece310010-bib-0059] Oakwood, M. , Bradley, A. J. , & Cockburn, A. (2001). Semelparity in a large marsupial. Proceedings of the Royal Society of London B: Biological Sciences, 268, 407–411. 10.1098/rspb.2000.1369 PMC1088621 11270438

[ece310010-bib-0060] Oli, M. K. , & Dobson, F. S. (2003). The relative importance of life‐history variables to population growth rate in mammals: Cole's predictions revisited. American Naturalist, 161, 422–440. 10.1086/367591 12699222

[ece310010-bib-0061] Owen‐Smith, N. , & Mason, D. R. (2005). Comparative changes in adult vs. juvenile survival affecting population trends of African ungulates. Journal of Animal Ecology, 74, 762–773. 10.1111/j.1365-2656.2005.00973.x

[ece310010-bib-0062] Paradis, E. , Baillie, S. R. , Sutherland, W. J. , & Gregory, R. D. (2002). Exploring density‐dependent relationships in demographic parameters in populations of birds at a large spatial scale. Oikos, 97, 293–307. 10.1034/j.1600-0706.2002.970215.x

[ece310010-bib-0063] Pardo, D. , Forcada, J. , Wood, A. G. , Tuck, G. N. , Ireland, L. , Pradel, R. , Croxall, J. P. , & Phillips, R. A. (2017). Additive effects of climate and fisheries drive ongoing declines in multiple albatross species. Proceedings of the National Academy of Sciences of the USA, 114, E10829–E10837. 10.1073/pnas.1618819114 29158390 PMC5740610

[ece310010-bib-0064] Ponciano, J. M. , Taper, M. L. , & Dennis, B. (2018). Ecological change points: the strength of density dependence and the loss of history. Theoretical Population Biology, 121, 45–59. 10.1016/j.tpb.2018.04.002 29705062 PMC5960640

[ece310010-bib-0065] Reed, D. H. , O'Grady, J. J. , Ballou, J. D. , & Frankham, R. (2003). The frequency and severity of catastrophic die‐offs in vertebrates. Animal Conservation, 6, 109–114. 10.1017/S1367943003147

[ece310010-bib-0066] Ricker, W. E. (1954). Stock and recruitment. Journal of the Fisheries Research Board of Canada, 11, 559–623. 10.1139/f54-039

[ece310010-bib-0067] Ricker, W. E. (1958). Handbook of computations for biological statistics of fish populations. Fisheries Research Board of Canada.

[ece310010-bib-0068] Rueda‐Cediel, P. , Anderson, K. E. , Regan, T. J. , Franklin, J. , & Regan, H. M. (2015). Combined influences of model choice, data quality, and data quantity when estimating population trends. PLoS One, 10, e0132255. 10.1371/journal.pone.0132255 26177511 PMC4503393

[ece310010-bib-0069] Sæther, B.‐E. , & Bakke, Ø. (2000). Avian life history variation and contribution of demographic traits to the population growth rate. Ecology, 81, 642–653. 10.1890/0012-9658(2000)081[0642:ALHVAC]2.0.CO;2

[ece310010-bib-0070] Sæther, B.‐E. , Engen, S. , & Matthysen, E. (2002). Demographic characteristics and population dynamical patterns of solitary birds. Science, 295, 2070–2073. 10.1126/science.1068766 11896278

[ece310010-bib-0071] Sinclair, A. R. E. , & Pech, R. P. (1996). Density dependence, stochasticity, compensation and predator regulation. Oikos, 75, 164–173. 10.2307/3546240

[ece310010-bib-0072] Smith, S. H. (1935). The role of biotic factors in the determination of population densities. Journal of Economic Entomology, 28, 873–898. 10.1093/jee/28.6.873

[ece310010-bib-0073] Sugiura, N. (1978). Further analysis of the data by Akaike's information criterion and the finite corrections. Communications in Statistics, Theory and Methods, A7, 13–26. 10.1080/03610927808827599

[ece310010-bib-0074] Thibaut, L. M. , & Connolly, S. R. (2020). Hierarchical modeling strengthens evidence for density dependence in observational time series of population dynamics. Ecology, 101, e02893. 10.1002/ecy.2893 31529700

[ece310010-bib-0075] Thorson, J. T. , Skaug, H. J. , Kristensen, K. , Shelton, A. O. , Ward, E. J. , Harms, J. H. , & Benante, J. A. (2015). The importance of spatial models for estimating the strength of density dependence. Ecology, 96, 1202–1212. 10.1890/14-0739.1 26236835

[ece310010-bib-0076] Traill, L. W. , Brook, B. W. , Frankham, R. , & Bradshaw, C. J. A. (2010). Pragmatic population viability targets in a rapidly changing world. Biological Conservation, 143, 28–34. 10.1016/j.biocon.2009.09.001

[ece310010-bib-0077] Turchin, P. (2003). Complex population dynamics: a theoretical/empirical synthesis. Princeton University Press.

[ece310010-bib-0078] Verhulst, P. F. (1838). Notice sur la loi que la population poursuit dans son accroissement. Correspondance mathématique et Physique, 10, 113–121.

[ece310010-bib-0079] Wu, Z. , Huang, N. E. , Long, S. R. , & Peng, C.‐K. (2007). On the trend, detrending, and variability of nonlinear and nonstationary time series. Proceedings of the National Academy of Sciences of the USA, 104, 14889–14894. 10.1073/pnas.0701020104 17846430 PMC1986583

