# Supplementary material for: Logistic‐growth models measuring density feedback are sensitive to population declines, but not fluctuating carrying capacity
Source: Ecol Evol. 2023 Apr 26;13(4):e10010. doi: 10.1002/ece3.10010 (PMC10131297; doi:10.1002/ece3.10010)
Supplement: Supplementary file 1 — Appendix S1 [file ECE3-13-e10010-s001.docx]

**Supporting Information**

**Logistic-growth models measuring density feedback are sensitive to population declines, but not fluctuating carrying capacity**

**Appendix 1 Confirming simulated populations were nonstationary**

The focus of our study is measuring how much nonstationarity drives decoupling of component *versus* ensemble compensatory density feedbacks in 10,000 simulated abundance time series for each of 21 test species and 9 demographic scenarios. We quantify the magnitude of stationarity (measured as return rates ${\bar{T}_{R}}/{\text{Var}\left( T_{\text{R}} \right)}$ to carrying capacity, so higher return rates imply higher stationarity; Section 2.5) imposed to the simulated series (Section 2.6) per scenario (Section 2.7) incorporating stochastic survival rates (Scenario *i*), catastrophic (*ii* and *iii*) and harvest (*iv* and *v*) mortality, varying carrying capacity (*vi* to *viii*) and lack of component density feedback on survival (scenario *ix*). Scenarios *i* to *viii* all embodied a component density feedback on survival.

Scenario *i* (stochastic survival rates with fixed carrying capacity) resulted in the highest stationarity among scenarios with a median return rate of ~ 0.28 (interquartile range: 0.17–0.43) across 21,000 simulated time series (10,000 series × 21 test species) (Fig. S1). Adding generationally scaled 50% catastrophic (density-independent) mortality reduced stationarity by ~50% to a median return rate of ~ 0.08 (0.05–0.16) (Scenario *ii*) (Fig. S1).

Figure S1. Truncated violin plots showing the distribution of the stationarity index (return rate) ${\bar{T}_{R}}/{\text{Var}\left( T_{\text{R}} \right)}$ (where $\bar{T}_{R}$ >> $\text{Var}\left( T_{\text{R}} \right)$ implies high stationarity) across 10,000 times series of population abundance per species for 21 test species (list in Table 2) obtained from age-structured populations subjected to a compensatory component density feedback on survival over 40 generations — without (Scenario *i*; pink; left) and with (Scenario *ii*; dark grey; right) catastrophic mortality. Each scenario includes 21,000 simulated time series of abundance (10,000 per species, Table 2).


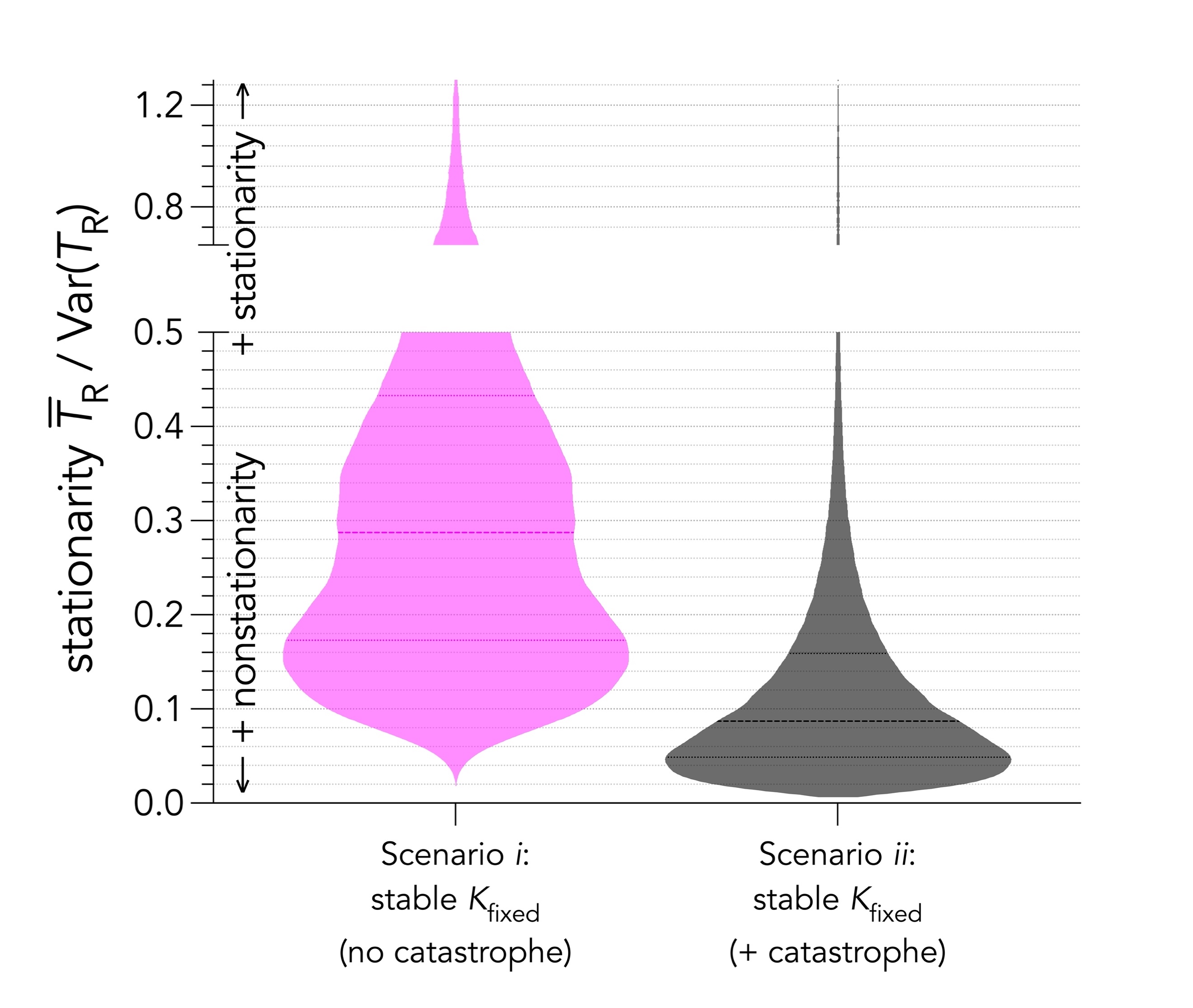


A similar reduction occurred for scenarios with pulse catastrophic (Scenario *iii*: median return rate = 0.05 [0.03–0.09]) and harvest (Scenarios *iv*: median return rate = 0.06 [0.03–0.10]; Scenario *v*: median return rate = 0.06 [0.03–0.10]) mortality (Fig. S2) ⎯ all with fixed carrying capacities.

Figure S2. Truncated violin plots showing the distribution of the stationarity index (return rate) ${\bar{T}_{R}}/{\text{Var}\left( T_{\text{R}} \right)}$ ($\bar{T}_{R}$ >> $\text{Var}\left( T_{\text{R}} \right)$ implies high stationarity) across 10,000 times series of population abundance per species for 21 test species (list in Table 2) obtained from age-structured populations subjected to a compensatory component density feedback on survival over 40 generations — a pulse disturbance of 90% mortality at the first 20 generations (20*G*; green; Scenario *iii*), weakly declining (*r* ≅ -0.001; red; Scenario *iv*), and strongly declining (*r* ≅ -0.01; blue; Scenario *v*). Each scenario includes 21,000 simulated time series of abundance (10,000 per species, Table 2).


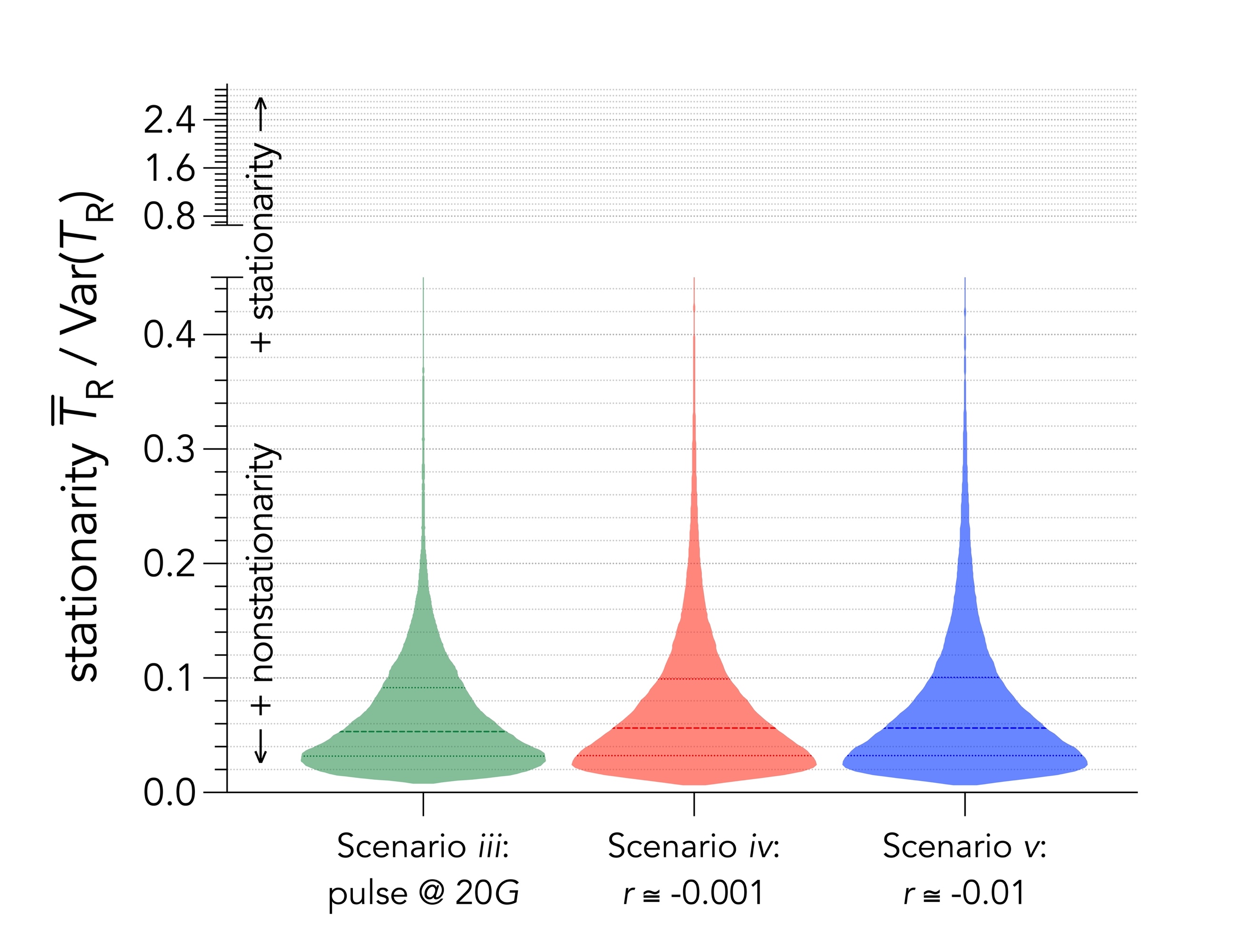


The scenarios emulating non-declining fluctuations in carrying capacity (Scenarios *vi* and *vii*) increased stationarity (relative to Scenario *i* with fixed *K*, Fig. S1) up to a median return rate of 0.14 (0.07–0.25) and 0.16 (0.08–0.29), respectively (Fig. S3). Only when the stochastic carrying capacity was forced to decline (Scenario *viii*), the time series became highly nonstationary with a median return rate of 0.05 (0.03–0.08) (Fig. S3). In the scenario lacking a component density feedback on survival (Scenario *ix*), stationarity was similar to that of scenario *i* with a median return rate 0.09 (0.06–0.14) (Fig. S3). Clearly, density-dependent mortality and declining carrying capacities are major drivers of nonstationarity in time series of population abundance.

Figure S3. Truncated violin plots showing the distribution of the stationarity index (return rate) ${\bar{T}_{R}}/{\text{Var}\left( T_{\text{R}} \right)}$ ($\bar{T}_{R}$ >> $\text{Var}\left( T_{\text{R}} \right)$ implies high stationarity) across 10,000 times series of population abundance per species for 21 test species (list in Table 2) obtained from age-structured populations subjected to a compensatory component density feedback on survival over 40 generations — stable projections with *K* varying stochastically (*K*_stoch_) around a constant mean with a constant variance (light blue; Scenario *vi*), *K* varying stochastically with a constant mean and an increasing variance (*K*_stoch_↑Var; gold; Scenario *vii*), *K* varying stochastically with a declining mean and a constant variance (↓*K*_stoch_; rust; Scenario *viii*), and an increase in the probability of 50% catastrophic (density-independent) mortality to produce stable population growth rates around 0 (no component density feedback; dark grey; Scenario *ix*). Each scenario includes 21,000 simulated time series of abundance (10,000 for each of 21 test species, Table 2).

**
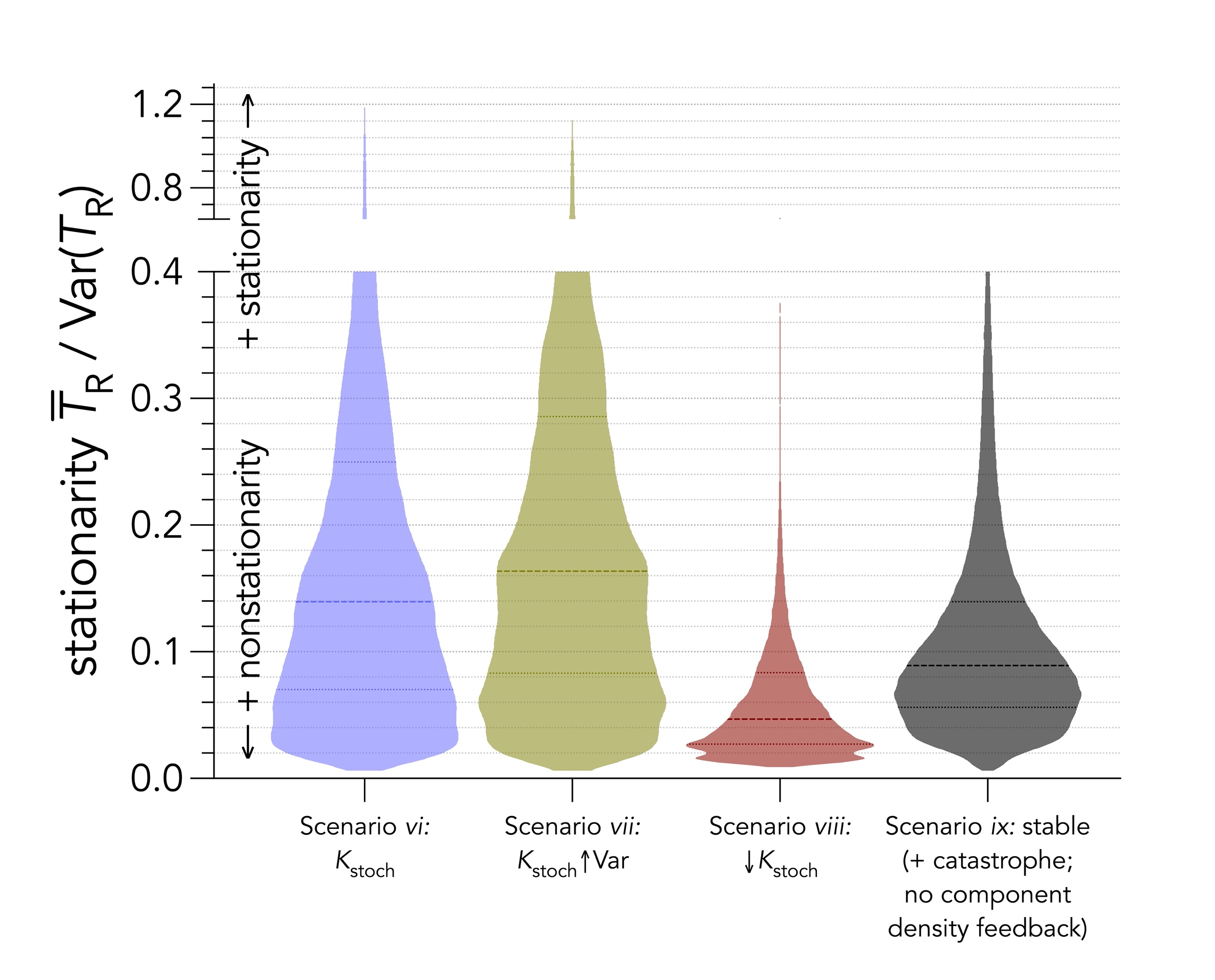
**

**Appendix 2 Effect of increasing standard deviation on survival for juveniles**

To test for potential effects of increased standard deviation on survival for juveniles relative to adults (Barraquand & Yoccoz, 2013; Hilde et al., 2020), we re-ran the 8 scenarios for the largest species considered (*Diprotodon optatum*) where we set the standard deviation in survival to three times that of adults for the initial age class (0.15), then decreased this value linearly to 0.05 by age at first reproduction (the value we used in the initial models for all age classes). Figure S4 shows clearly that adding additional variance in survival for juveniles makes almost no difference to the estimate of phenomenological feedback strength from the Gompertz models (the relationship is nearly 1:1, and statistically indistinguishable from a perfect match). Therefore, there is no need to add additional variance to the other species because this additional complexity would not alter our results.


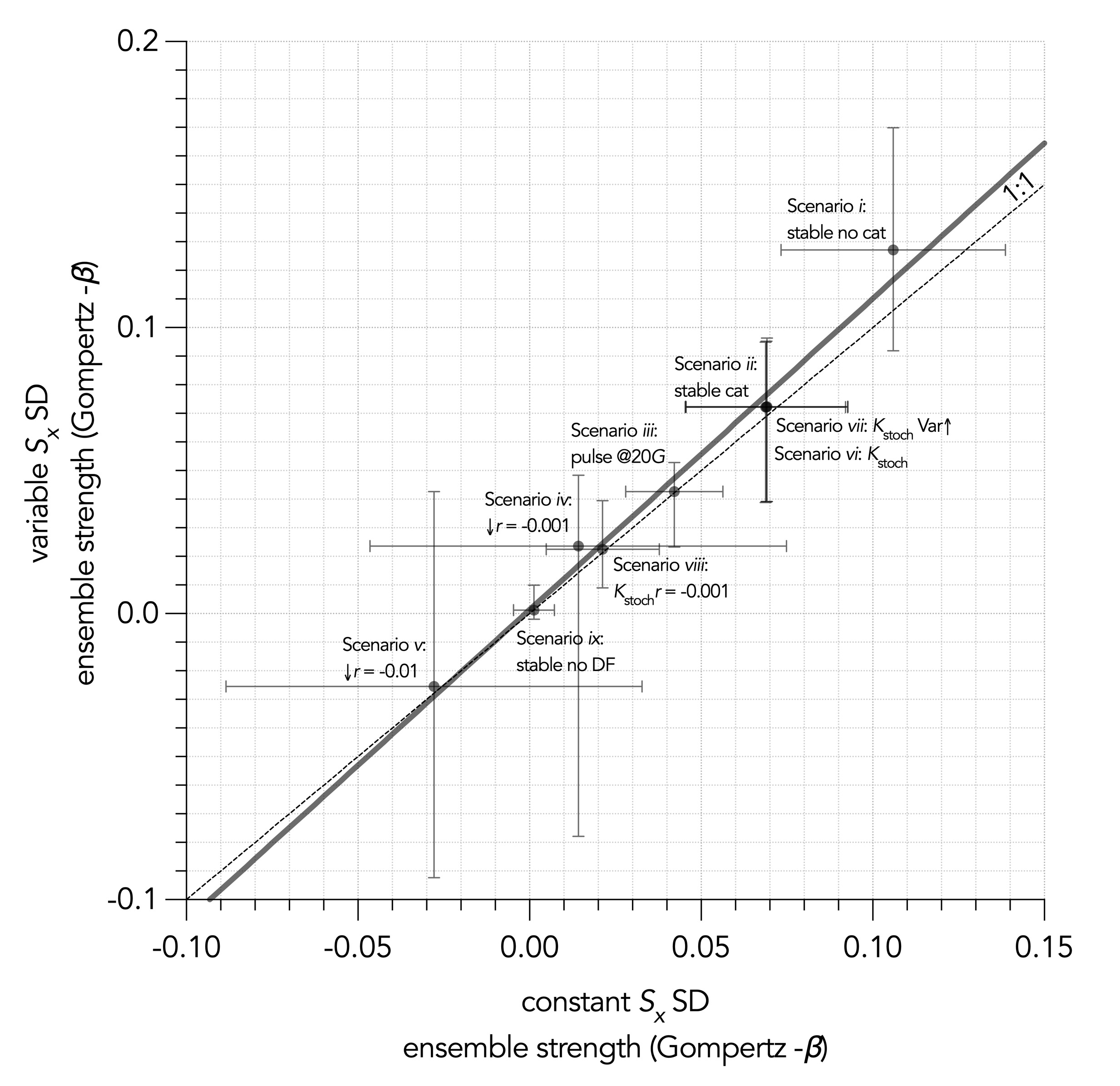
Figure S4 Comparison of ensemble feedback strength between models for the marsupial *Diprotodon optatum* across all scenarios where we kept the standard deviation in survival constant across age classes (0.05), or increased it for the juvenile age classes. The *x*-axis represents the strength of the (Gompertz) density feedback strengths as estimated from the phenomenological models under the assumption of constant variance in survival probability for each scenario (**Scenario *i*:** **stable no cat**: stable population size with no generationally scaled catastrophes; **Scenario *ii*:** **stable cat**: stable population size with generationally scaled catastrophes; **Scenario *iii*: pulse @ 20*G***: pulse disturbance at 20 generations; **Scenario *iv*:** ***r* = -0.001**: declining population size at *r* = -0.001; **Scenario *v*:** ***r* = -0.01**: declining population size at *r* = -0.01; **Scenario *vi*: *K*_stoch_**: stochastic carrying capacity (constant variance); **Scenario *vii*: *K*_stoch_ Var↑**: stochastic carrying capacity (increasing variance, but average, long-term *K* stable); **Scenario *viii*: *K*_stoch_ *r* = -0.001**: stochastic carrying capacity (increasing variance); **Scenario *ix*: stable no DF**: no density feedback, but catastrophe frequency/magnitude high enough to ensure average long-term stability ⎯ see Table 3). The *y*-axis represents the same estimates for the models assuming an increase in standard deviation in survival among juveniles. The 1:1 relationship is shown as a dashed line, demonstrating no effect on estimated the estimated strength of ensemble density feedback of increased standard deviation in juvenile survival relative to adults.

**Appendix 3 Component density feedbacks on fertility *versus* survival**

*Life history*

Based on previous work, we can expect density feedbacks to operate on survival more strongly than on fertility in *K*-selected species (our set of species, Table 2), and we can expect the opposite for *r*-selected species (Herrando-Pérez et al., 2012). Regardless, the main issue is that *reproductive success* and *reproduction* are often used synonymously in the literature, when the former commonly includes/confounds neonate/juvenile survival (i.e., whether the offspring survived). This is adequately accounted for in our models where the 0–1 year survival is specifically provided. In the cases of depressed maternal condition at high densities in large herbivores (Bonenfant et al., 2009), this is usually expressed as a reduction in neonate survival because of inadequate transfer of maternal resources post-partum. In cases where the proportion of lactating females in a population is negatively correlated with density (Bonenfant et al., 2009), the early loss of a neonate will precipitate the cessation of lactation in the mother. A similar pattern emerges when measuring the ratio of youngster:female numbers. In other words, it is not an effect of density on reproduction *per se*; rather, it mainly affects juvenile survival.

Conversely, variation in parameters such as inter-birth interval, rate of twinning rate, and age at primiparity are most related to reproduction *sensu stricto*. However, it is also essential to recognise that using data measured on large placental herbivores to infer processes of density feedbacks in marsupial reproduction is problematic for several reasons. First, most marsupials have a relatively fixed reproduction (compared to placentals), obligately producing 1 or 2 offspring per breeding cycle (e.g., koalas, wombats) (Fisher et al., 2001). Others like the Tasmanian devil produce nearly 40 neonates, but can only wean 4 successfully given the presence of 4 teats on the female. In fact, most variation in litter size among marsupials can be explained by body mass and diet type (Fisher et al., 2001). Marsupials therefore tend to adapt to environmental stressors effectively by discarding underdeveloped neonates to suit the rate of incoming resources (Morton et al., 1982). Because of the completely different developmental processes of the young *ex utero* in marsupials compared to placentals, reducing reproductive output requires little resource loss (Low, 1978). On the other hand, placentals do not really have this option because they rarely abort when they are stressed.

Second, many marsupials have relatively fixed life-history schedules compared to placentals, especially in the semelparous and semi-semelparous dasyuridae (we included three in our simulations: thylacines, devils, quolls) that have fixed longevities characterised by catastrophic end-of-life mortality (Bradshaw & Brook, 2005; Fisher et al., 2006).

All but five of our 21 test species (Table 2) we simulated are marsupials: the others include two monotremes (echidna, platypus) and three large birds (*Genyornis*, emu, brush turkey). Ironically, the long-lived monotremes potentially come the closest to inferences of density feedback on reproduction derived from placentals. Both female echidnas and platypuses have variable age at primiparity and breeding frequency (inter-birth interval) that could be related to resource availability/habitat type (Temple-Smith & Grant, 2002); however, there is no available evidence relating these reproductive characteristics to population density.

Neither is the evidence for density feedback on reproduction in birds unequivocal. For the same reason that reproductive output is often confounded with reproduction in large placental herbivores, egg production too is widely confounded with clutch size in birds, the latter being a product of the former along with hatchling survival. While there is ample evidence for clutch size–population density relationships in some passerines (Both, 1998; Both et al., 2000), there is no evidence for density feedback in clutch size in others (Torok & Toth, 1988) (Hario & Rintala, 2006), or the evidence is equivocal (Both, 2000; Dhondt et al., 1992). Emu (and by proxy, *Genyornis*). While clutch size can vary considerably, the resultant hatching success has more to do with male’s incubation capacity than the number of eggs produced by the female (Ryeland et al., 2021).

Given the ample evidence that density feedback on survival (at all stages) is the dominant process, and that we are relying on species with relatively inflexible reproduction compared to placentals, we argue that our approach with our 21 test species chosen (Table 2) therefore represents an *advantage* for the purposes of testing density-feedback hypotheses, because of the simpler and realistic assumptions regarding density feedback operating primarily on a single (but age-specific) life-history parameter.

*Modelling*

Even ignoring these arguments above, there is an operational question about how to combine the component signals from two interacting demographic rates. We cannot simply add the Gompertz slopes of the two feedback functions (one for survival; one for fertility) because they are expressed in different units. Neither can we standardise the coefficients (e.g., $\hat{\beta}/{\hat{SE}_{\beta}}$) and sum these because then we would lose the relative weighting of feedback strength (e.g., feedback in survival being more influential than feedback in fertility). We struggled with this operational dilemma during the construction of the initial models, which was yet another reason we decided to limit the component feedback signal to survival. Even if we could solve the mathematical dilemma described above, we are still setting the base stochastic models to long-term average stability over the projection interval (40 generations) by adjusting the feedback mechanism(s) accordingly. Thus, whether the component signal is embedded entirely within one demographic rate, or split into two (or more) functions, is mathematically redundant and the projection outcomes remain the same.

To demonstrate that including several feedback functions makes no difference to our conclusions, we re-ran Scenario *i* (Stochastic mortality, no catastrophic mortality, stable carrying capacity; see Table 3) for the marsupial *Diprotodon optatum*, but added a feedback function on fertility in addition to the one applied to survival, adjusting the functions accordingly so that average, long-term *r* = 0. In this particular example, fertility is forced to decline approximately 4%, and survival by 6%, as carrying capacity is achieved (following the relationships expressed by equation 1 in the main text:

$$\frac{a}{1+\left( \frac{\sum\text{n}}{b} \right)^{c}}$$

where for fertility: *a* = 1.00001; *b* = 245072.1; *c* = 1.556093, and for survival: *a* = 0.9996571; *b* = 158166.6.1; *c* = 1.775586).

Ignoring the problem of how to combine the two functions (i.e., density feedback on fertility *versus* survival, see above) into a single measure of component strength, the resultant median strength of the phenomenological Gompertz model for this example is 0.112 (95% confidence interval: 0.083–0.150). Compared to the same model with only a component density feedback on survival (as reported in the main text), the Gompertz strength was 0.106 (0.077–0.143). The lack of any appreciable effect on the estimated ensemble feedback therefore justifies restricting our analyses to a component feedback on survival.

Figure S5 Probability of an ensemble compensatory density-feedback signal (Pr(*density feedback*) = Σ*w*AIC*_c_*-*density feedback* = sum of Akaike’s information criterion weights across the Ricker- and Gompertz-logistic models relative to two density-independent models (random and exponential population growth) — see Methods) in abundance time series for simulated populations of 21 long-lived species of Australian mammals and birds (list in Table 2) subjected to compensatory density feedback on survival and experiencing 50% catastrophic (density-independent) mortality over 40 generations. Each probability surface represents one of the 21 test species (Table 2), so plots show the overlapping median probability density over 10,000 times series of abundance per species and for each of four demographic scenarios (detailed in Table 3), including (**a**) a carrying capacity is fixed (*K*_fixed_) with 50% catastrophic (density-independent) mortality (Scenario *ii*), (**b**) a pulse disturbance of 90% mortality at 20 generations (20*G*; Scenario *iii*), and (**c**) weakly declining ($\bar{r}$ ≅ -0.001; Scenario *iv*), and (d) strongly declining ($\bar{r}$ ≅ -0.01; Scenario *v*) populations ⎯ scenarios summarized in Table 3. See Fig. S8 for bootstrapped mean Spearman correlation coefficients for each scenario.


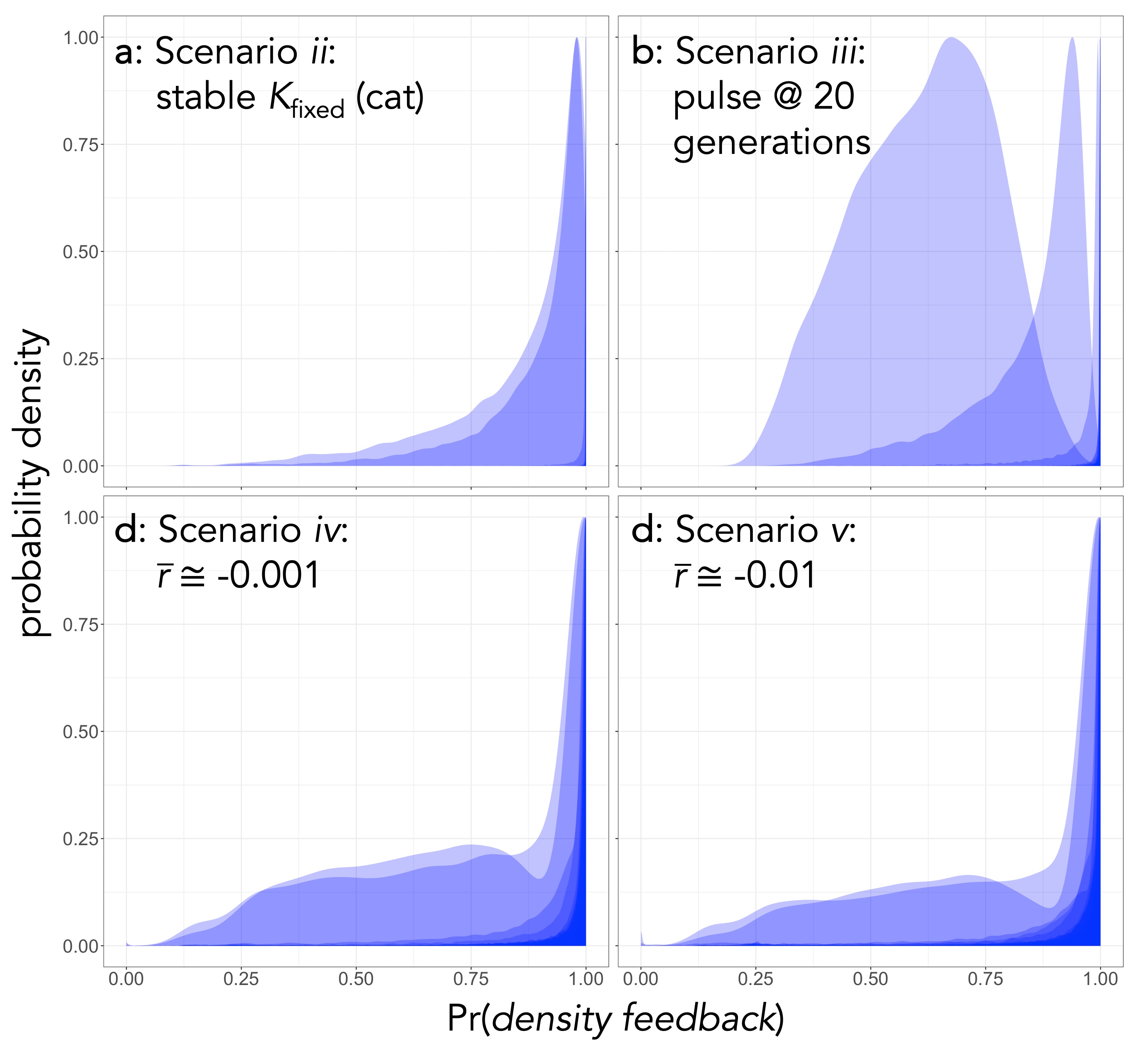


Figure S6 Probability of an ensemble compensatory density-feedback signal (Pr(*density feedback*) = Σ*w*AIC*_c_*-*density feedback* = sum of Akaike’s information criterion weights across the Ricker- and Gompertz-logistic models relative to two density-independent models (random and exponential population growth) — see Methods) in abundance time series for simulated populations of 21 long-lived species of Australian mammals and birds (list in Table 2) subjected to compensatory density feedback on survival and experiencing fluctuations in carrying capacity (*K*) along with 50 % catastrophic (density-independent) mortality over 40 generations. Each probability surface represents one of the 21 test species (Table 2), so plots show the overlapping median probability density over 10,000 times series of abundance per species and for each of four demographic scenarios (detailed in Table 2), including (**a**) a stable demographic projection where *K* is fixed (*K*_fixed_) (Scenario *ii*), (**b**) *K* varies stochastically (*K*_stoch_) around a constant mean with a constant variance (Scenario *vi*), (**c**) *K* varying stochastically with a constant mean and increasing variance (*K*_stoch_↑Var; Scenario *vii*), and (**d**) *K* varying stochastically with a declining mean and a constant variance (↓*K*_stoch_; Scenario *viii*) ⎯ scenarios summarized in Table 3. See Fig. S8 for bootstrapped mean Spearman correlation coefficients for each scenario.

**
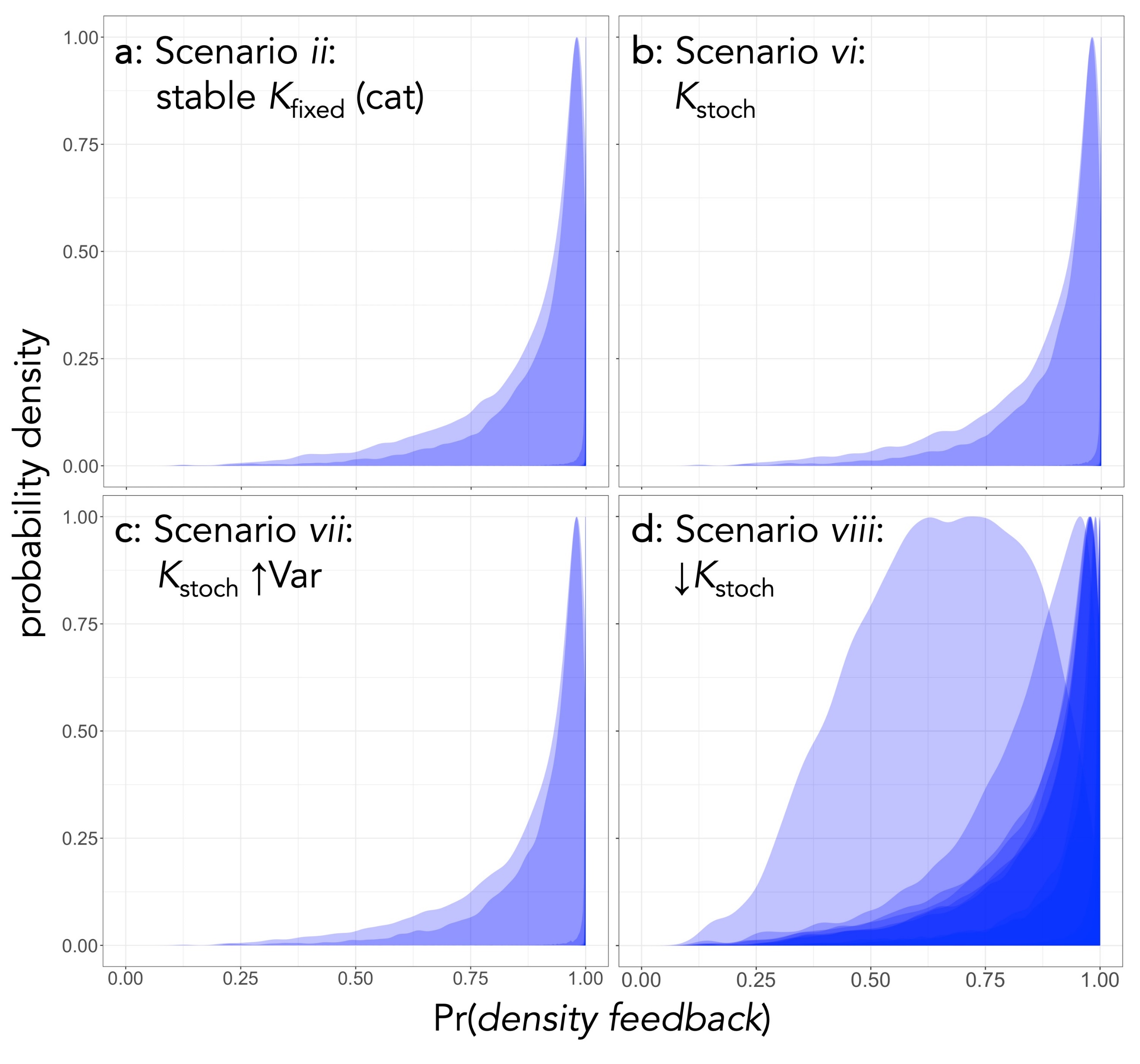
**

Figure S7 Bootstrapped mean (with 80 % confidence intervals; 100,000 resamples) probability of an ensemble compensatory density-feedback signal (Pr(*density feedback*) = Σ*w*AIC*_c_*-*density feedback* = sum of Akaike’s information criterion weights across the Ricker- and Gompertz-logistic models relative to two density-independent models (random and exponential population growth) — see Methods) in abundance time series for simulated populations of 21 long-lived species of Australian mammals and birds for populations (list in Table 2) subjected to compensatory density feedback on survival and experiencing fluctuations in carrying capacity (*K*) and/or 50% catastrophic (density-independent) mortality (scenarios detailed in Table 3). Demographic scenarios include (**a**) *K* fixed with no catastrophic mortality (Scenario *i*: *K*_fixed_ no cat), and (**b**) with catastrophic mortality (Scenario *ii*: *K*_fixed_ cat), (**c**) a pulse disturbance of 90% mortality at 20 generations (Scenario *iii*), (**d**) weakly declining (Scenario *iv*: $\bar{r}$ ≅ -0.001) and (**e**) strongly declining (Scenario *v*: $\bar{r}$ ≅ -0.01) populations, (**f**) *K* varying stochastically(*K*_stoch_) around a constant mean with a constant variance (Scenario *vi*), (**g**) *K* varying stochastically with a constant mean and increasing variance (Scenario *vii*: *K*_stoch_↑Var), and (**h**) *K* varying stochastically with a declining mean and a constant variance (scenario *viii*: ↓*K*_stoch_) ⎯ scenarios summarized in Table 3. The vertical dashed line at Pr(*density feedback*) = 0.5 in each panel is the point below which the evidence for a density-independent model [Pr(*density independence*) = Σ*w*AIC*_c_*-*density independence* = sum of Akaike’s information criterion weights across the random walk and exponential models] is greater than Pr(*density feedback*). See Table 2 for species abbreviations along the y-axes.


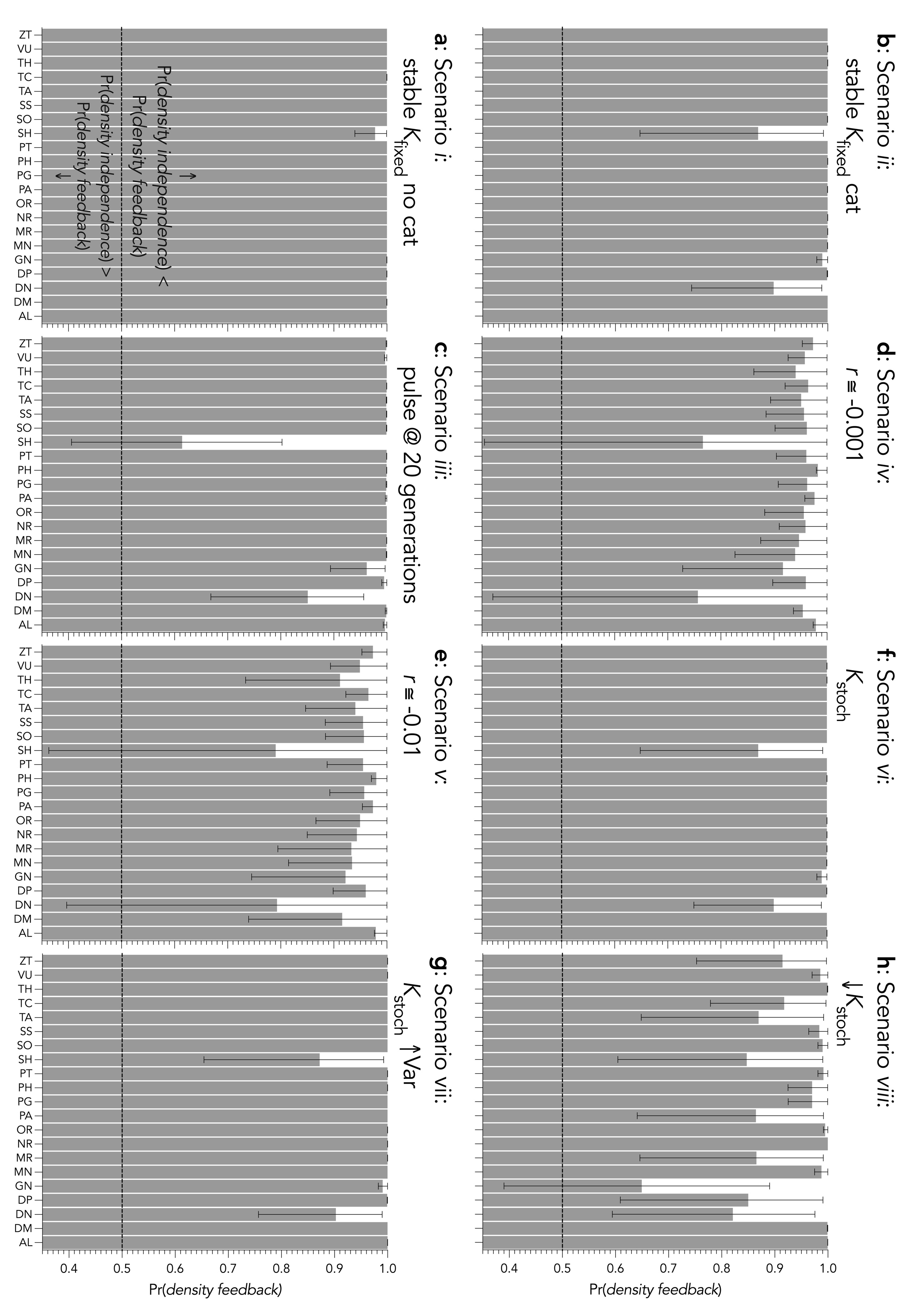


Figure S8 Bootstrapped (10,000 iterations) Spearman’s correlation *ρ* between (**a**) ensemble density feedback strength (Gompertz slope -*β*, the reduction of survival as population density increases) *and* component feedback strength on survival (1 – *S*_red_, the reduction in survival as population density increases), and (**b**) ensemble feedback strength *and* the stationarity metric (return rate) ${\bar{T}_{R}}/{\text{Var}\left( T_{\text{R}} \right)}$ for 10,000 simulated populations across each of 21 long-lived species of Australian mammals and birds for populations (list in Table 2) subjected to compensatory density feedback on survival and experiencing fluctuations in carrying capacity (*K*) and/or 50 % catastrophic (density-independent) mortality (scenarios detailed in Table 3) . Demographic scenarios include *K* fixed (*K*_fixed_) with no catastrophic mortality (no cat; Scenario *i*), and catastrophic mortality in combination with *K*_fixed_ (cat; Scenario *ii*), a pulse disturbance of 90% mortality at 20 generations (Scenario *iii*), weakly declining ($\bar{r}$ ≅ -0.001; Scenario *iv*) and (**E**) strongly declining ($\bar{r}$ ≅ -0.01; Scenario *v*) populations, *K* varying stochastically(*K*_stoch_) around a constant mean with a constant variance (Scenario *vi*), *K* varying stochastically with a constant mean and increasing variance (*K*_stoch_↑Var; Scenario *vii*), and *K* varying stochastically with a declining mean and a constant variance (↓*K*_stoch_; Scenario *viii*) ⎯ scenarios summarized in Table 3.

**
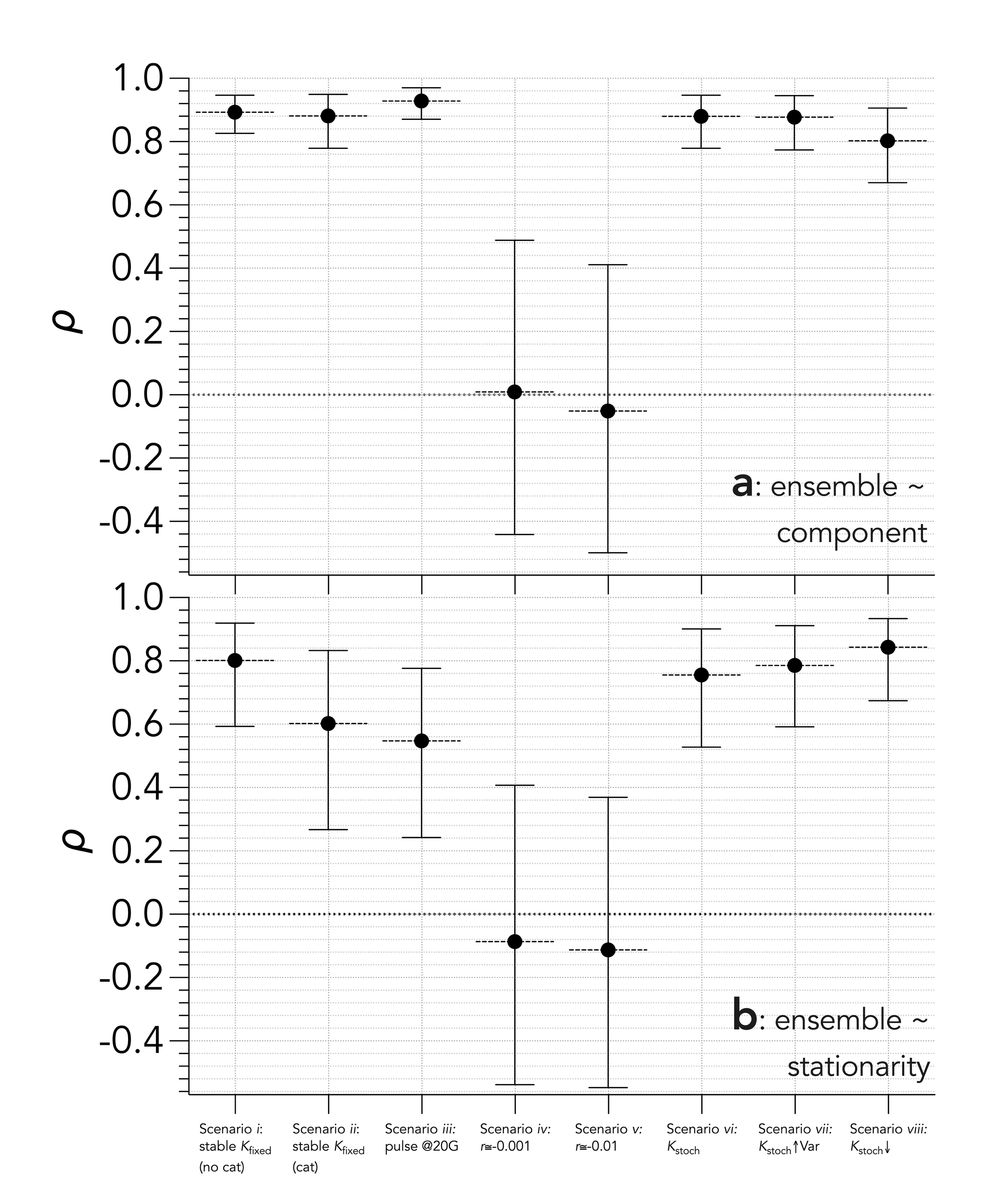
**

Figure S9 Truncated violin plots showing the distribution of the stationarity index (return rate) ${\bar{T}_{R}}/{\text{Var}\left( T_{\text{R}} \right)}$ across 10,000 time series of population abundance per species and all 21 species (see species list in Table 2) obtained from age-structured populations for scenarios showing carrying capacity fixed with component compensatory density-feedback on survival and 50% catastrophic (density-independent) mortality to produce stable population growth rates around 0 over 40 (Scenario *ii*; detailed in Table 3) and 120 generations (*G*)*.*

**
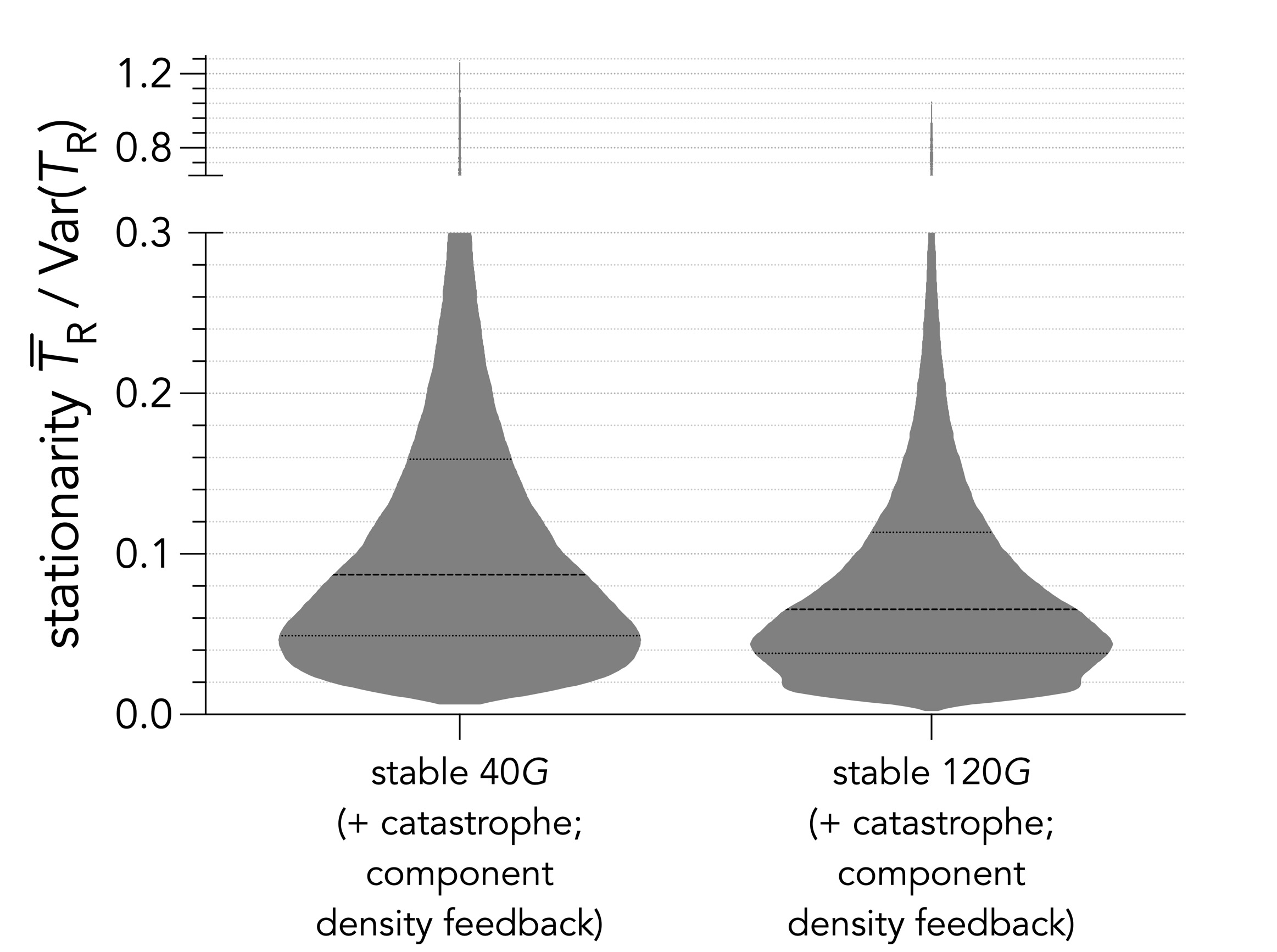
**

Figure S10 Relationship between strength of component density feedback and generation length (years) across 10,000 time series of population abundance for each of 21 test species (list in Table 2) obtained from age-structured populations subjected to a compensatory component density feedback on survival over 40 generations for a demographic scenario with constant carrying capacity and no catastrophic (density-independent) mortality (Scenario *i*; detailed in Table 3). The dashed grey line indicates a least-squares-fitted (adjusted coefficient of regression *R*^2^ = 0.58) exponential plateau model of the form: *y* = *y*_max_ - (*y*_max_ - *y*_0_)e^-^*^kG^*, where *y*_0_ = starting value of component strength, *y*_max_ = maximum component strength, *k* = rate constant (years^-1^) and *GL* = generation length (years). Species notation: DP = *Diprotodon optatum*, PA = *Palorchestes azael*, ZT = *Zygomaturus trilobus*, PH = *Phascolonus gigas*, VU *Vombatus ursinus* (herbivore vombatiform); PG = *Procoptodon goliah*, SS = *Sthenurus stirlingi*, PT = *Protemnodon anak*, SO = *Simosthenurus occidentalis*, MN = *Metasthenurus newtonae*, OR = *Osphranter rufus* (herbivore macropodiformes); GN = *Genyornis newtoni*, DN = *Dromaius novaehollandiae* (large omnivore birds) , AL = *Alectura lathami*; TC = *Thylacoleo carnifex*, TH = *Thylacinus cynocephalus*, SH = *Sarcophilus harrisii* (carnivores) , DM = *Dasyurus maculatus*; MR = *Megalibgwilia ramsayi*; TA = *Tachyglossus aculeatus* (invertivore monotremes).


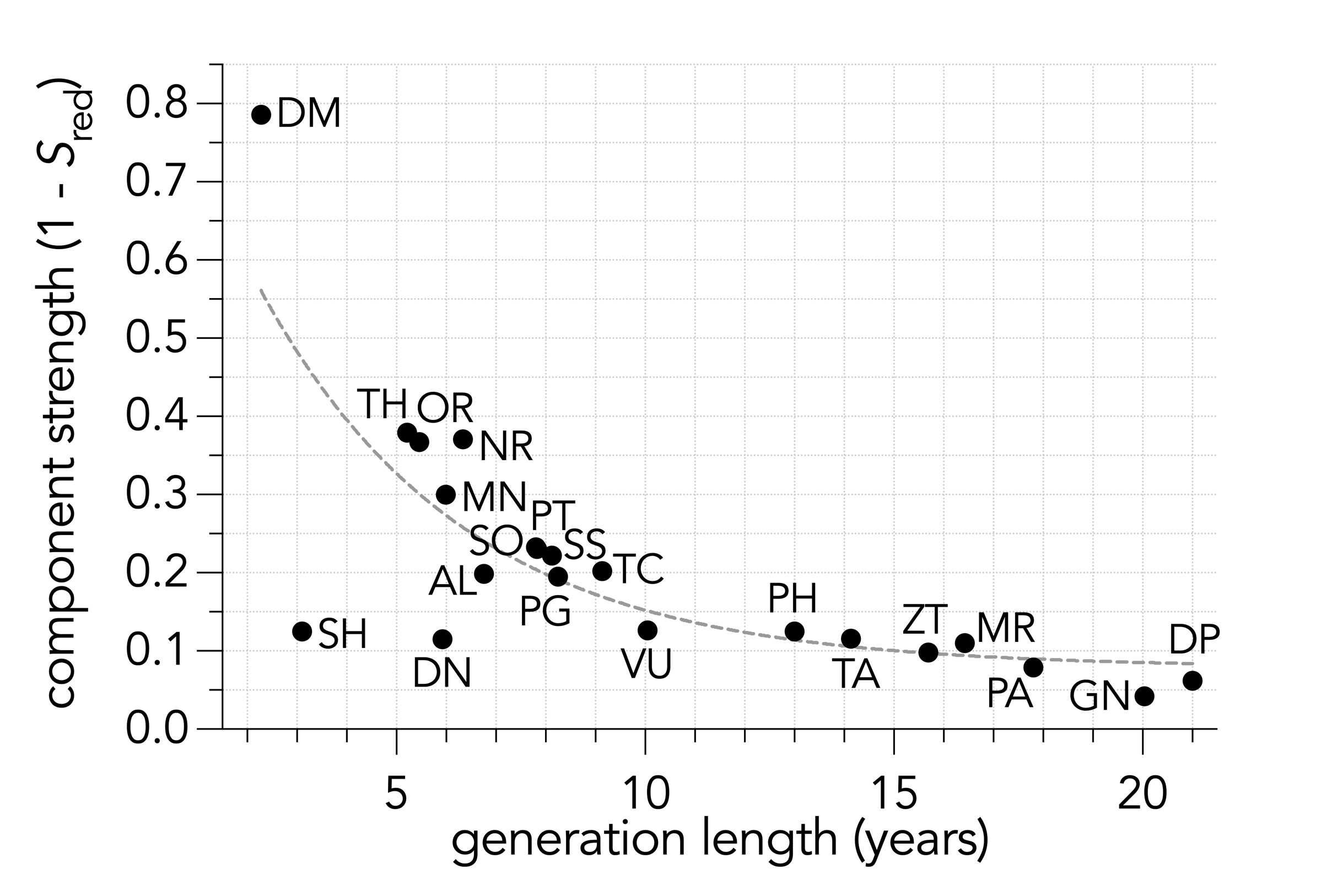


Figure S11 Relationships between the stationarity index (return rate) ${\bar{T}_{R}}/{\text{Var}\left( T_{\text{R}} \right)}$ and generation length across 10,000 times series of population abundance per species and all 21 test species (see list in Table 2) obtained from age-structured populations subjected to a compensatory component density feedback on survival over 40 generations, according to seven demographic scenarios (detailed in Table 3). Demographic scenarios include (**a**) carrying capacity *K* fixed (*K*_fixed_; Scenario *ii*), (**b**) a pulse disturbance of 90% mortality at 20 generations (20*G*; Scenario *iii*), (**c**) weakly declining ($\bar{r}$ ≅ -0.001; Scenario *iv*) and (**d**) strongly declining ($\bar{r}$ ≅ -0.01; Scenario *v*) populations, (**e**) *K* varying stochastically (*K*_stoch_) around a constant mean with a constant variance (Scenario *vi*), (**f**) *K* varying stochastically with a constant mean and increasing variance (*K*_stoch_↑Var; Scenario *vii*), and (**g**) *K* varying stochastically with a declining mean and a constant variance (↓*K*_stoch_; Scenario *viii*) ⎯ scenarios summarized in Table 3.


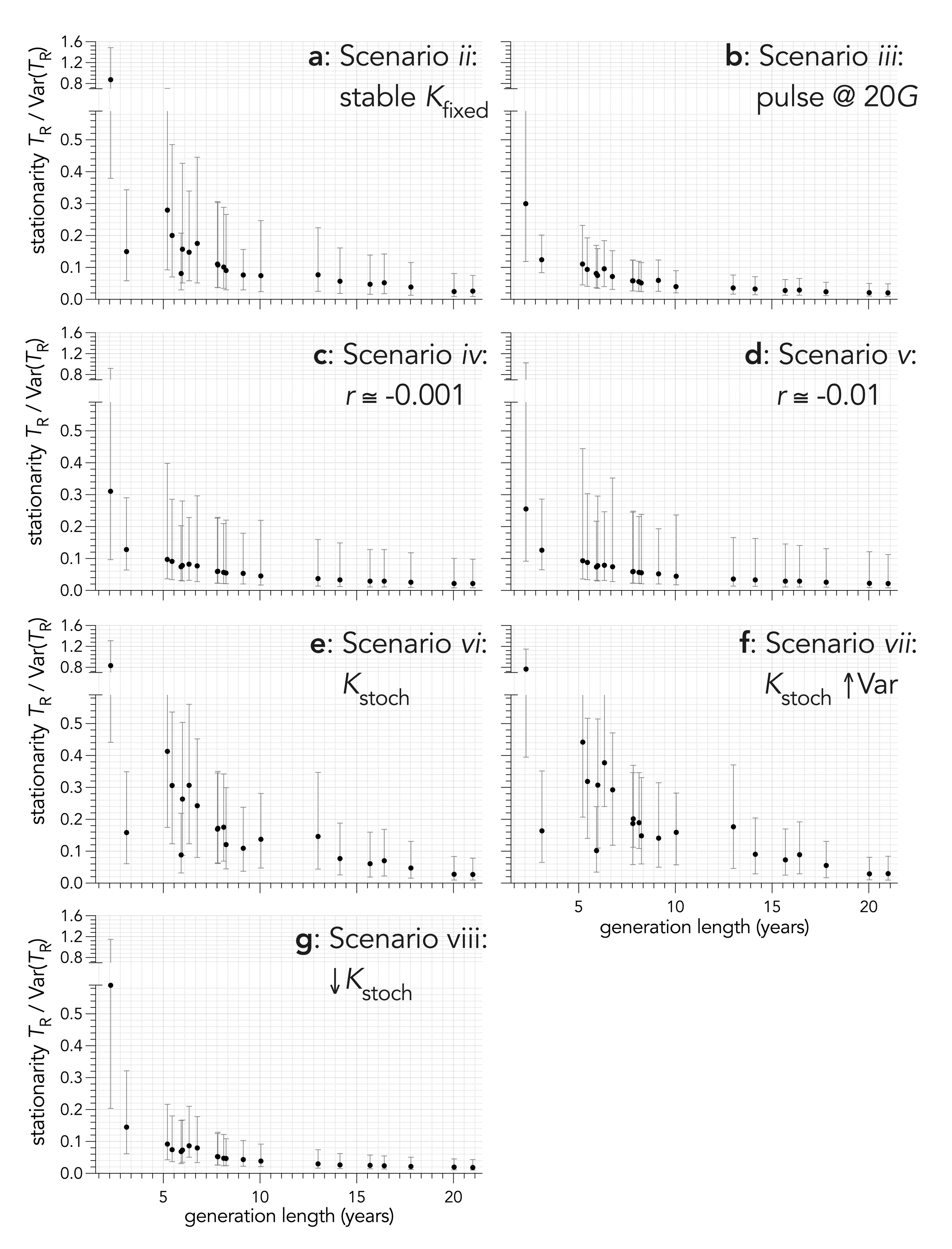


Figure S12 Relationships between the strength of ensemble (Gompertz slope -*β*, the reduction of survival as population density increases) and generation length across 10,000 times series of population abundance per species and all 21 test species (list in Table 2) obtained from age-structured populations subjected to a compensatory component density feedback on survival over 40 generations, according to seven demographic scenarios (detailed in Table 3). Demographic scenarios include (**a**) carrying capacity *K* fixed (*K*_fixed_; Scenario *ii*), (**b**) a pulse disturbance of 90% mortality at 20 generations (20*G*; Scenario *iii*), (**c**) weakly declining ($\bar{r}$ ≅ -0.001; Scenario *iv*) and (**d**) strongly declining ($\bar{r}$ ≅ -0.01; Scenario *v*) populations, (**e**) *K* varying stochastically (*K*_stoch_) around a constant mean with a constant variance (Scenario *vi*), (**f**) K varying stochastically with a constant mean and increasing variance (*K*_stoch_↑Var; Scenario *vii*), and (**g**) *K* varying stochastically with a declining mean and a constant variance (↓*K*_stoch_; Scenario *viii*) ⎯ scenarios summarized in Table 3.


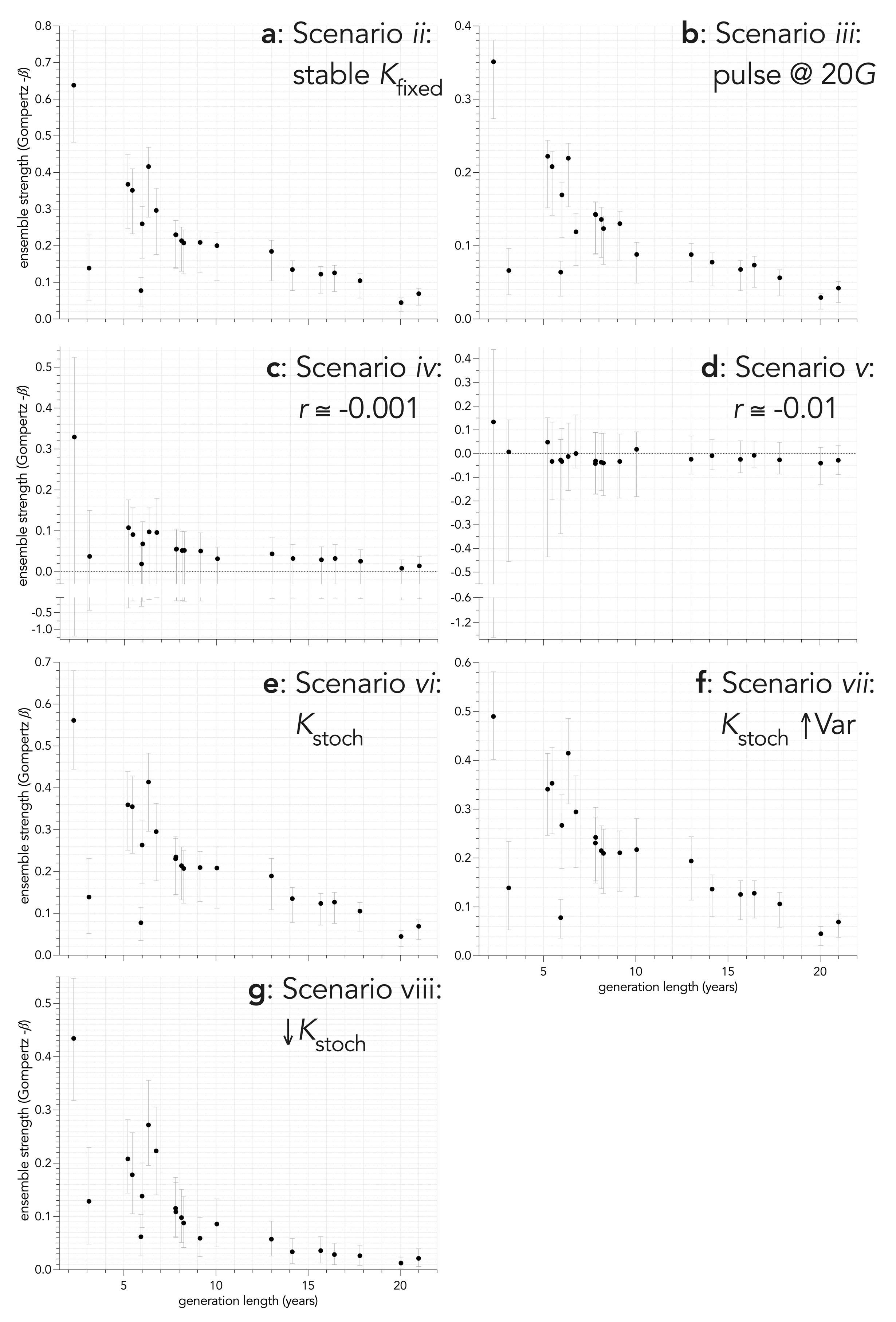


**Supporting information references**

Barraquand, F., & Yoccoz, N. G. (2013). When can environmental variability benefit population growth? Counterintuitive effects of nonlinearities in vital rates. *Theoretical Population Biology, 89*, 1-11. doi:10.1016/j.tpb.2013.07.002

Bonenfant, C., Gaillard, J. M., Coulson, T., Festa‐Bianchet, M., Loison, A., Garel, M., . . . Duncan, P. (2009). Chapter 5 Empirical evidence of density‐dependence in populations of large herbivores. In *Advances in Ecological Research* (Vol. 41, pp. 313-357): Academic Press.

Both, C. (1998). Density dependence of clutch size: habitat heterogeneity or individual adjustment? *Journal of Animal Ecology, 67*, 659-666. doi:10.1046/j.1365-2656.1998.00227.x

Both, C. (2000). Density dependence of avian clutch size in resident and migrant species: is there a constraint on the predictability of competitor density? *Journal of Avian Biology, 31*, 412-417. doi:10.1034/j.1600-048X.2000.310317.x

Both, C., Tinbergen, J. M., & Visser, M. E. (2000). Adaptive density dependence of avian clutch size. *Ecology, 81*, 3391-3403. doi:10.1890/0012-9658(2000)081[3391:ADDOAC]2.0.CO;2

Bradshaw, C. J. A., & Brook, B. W. (2005). Disease and the devil: density-dependent epidemiological processes explain historical population fluctuations in the Tasmanian devil. *Ecography, 28*, 181-190. doi:10.1111/j.0906-7590.2005.04088.x

Dhondt, A. A., Kempenaers, B., & Adriaensen, F. (1992). Density-dependent clutch size caused by habitat heterogeneity. *Journal of Animal Ecology, 61*, 643-648. doi:10.2307/5619

Fisher, D. O., Double, M. C., Blomberg, S. P., Jennions, M. D., & Cockburn, A. (2006). Post-mating sexual selection increases lifetime fitness of polyandrous females in the wild. *Nature, 444*, 89-92. doi:10.1038/nature05206

Fisher, D. O., Owens, I. P. F., & Johnson, C. N. (2001). The ecological basis of life history variation in marsupials. *Ecology, 82*, 3531-3540. doi:10.1890/0012-9658(2001)082[3531:TEBOLH]2.0.CO;2

Hario, M., & Rintala, J. (2006). Fledgling production and population trends in Finnish common eiders (*Somateria mollissima mollissima*) — evidence for density dependence. *Canadian Journal of Zoology, 84*, 1038-1046. doi:10.1139/z06-077

Herrando-Pérez, S., Delean, S., Brook, B. W., & Bradshaw, C. J. A. (2012). Strength of density feedback in census data increases from slow to fast life histories. *Ecology and Evolution, 2*, 1922-1934. doi:10.1002/ece3.298

Hilde, C. H., Gamelon, M., Sæther, B.-E., Gaillard, J.-M., Yoccoz, N. G., & Pélabon, C. (2020). The demographic buffering hypothesis: evidence and challenges. *Trends in Ecology and Evolution, 35*, 523-538. doi:10.1016/j.tree.2020.02.004

Low, B. S. (1978). Environmental uncertainty and the parental strategies of marsupials and placentals. *The American Naturalist, 112*, 197-213. doi:10.1086/283260

Morton, S. R., Recher, H. F., Thompson, S. D., & Braithwaite, R. W. (1982). Comments on the relative advantages of marsupial and eutherian reproduction. *American Naturalist, 120*, 128-134. doi:10.1086/283975

Ryeland, J., House, C. M., Umbers, K. D. L., & Spencer, R.-J. (2021). Optimal clutch size and male incubation investment in the male-only incubating emu (*Dromaius novaehollandiae*). *Behavioral Ecology and Sociobiology, 75*, 168. doi:10.1007/s00265-021-03110-4

Temple-Smith, P., & Grant, T. (2002). Uncertain breeding: a short history of reproduction in monotremes. *Reproduction, Fertility and Development, 13*, 487-497. doi:10.1071/RD01110

Torok, J., & Toth, L. (1988). Density dependence in reproduction of the collared flycatcher (*Ficedula albicollis*) at high population levels. *Journal of Animal Ecology, 57*, 251-258. doi:10.2307/4776
